# Determining the level of social distancing necessary to avoid future COVID-19 epidemic waves: a modelling study for North East London

**DOI:** 10.1038/s41598-021-84907-1

**Published:** 2021-03-11

**Authors:** Nathan Cheetham, William Waites, Irene Ebyarimpa, Werner Leber, Katie Brennan, Jasmina Panovska-Griffiths

**Affiliations:** 1 Financial Strategy Team, NHS North East London Commissioning Alliance, London, UK; 2 School of Informatics, University of Edinburgh, Edinburgh, UK; 3 Centre for Clinical Effectiveness and Health Data Science, Institute of Population Health Sciences, Barts School of Medicine and Dentistry, Queen Mary University of London, London, UK; 4 Department of Applied Health Care, Institute of Epidemiology & Health Care, University College London, London, UK; 5 Institute for Global Health, University College London, London, UK; 6 The Wolfson Centre for Mathematical Biology and The Queen’s College, University of Oxford, Oxford, UK

**Keywords:** Applied mathematics, Epidemiology, Viral infection

## Abstract

Determining the level of social distancing, quantified here as the reduction in daily number of social contacts per person, i.e. the daily contact rate, needed to maintain control of the COVID-19 epidemic and not exceed acute bed capacity in case of future epidemic waves, is important for future planning of relaxing of strict social distancing measures. This work uses mathematical modelling to simulate the levels of COVID-19 in North East London (NEL) and inform the level of social distancing necessary to protect the public and the healthcare demand from future COVID-19 waves. We used a Susceptible-Exposed-Infected-Removed (SEIR) model describing the transmission of SARS-CoV-2 in NEL, calibrated to data on hospitalised patients with confirmed COVID-19, hospital discharges and in-hospital deaths in NEL during the first epidemic wave. To account for the uncertainty in both the infectiousness period and the proportion of symptomatic infection, we simulated nine scenarios for different combinations of infectiousness period (1, 3 and 5 days) and proportion of symptomatic infection (70%, 50% and 25% of all infections). Across all scenarios, the calibrated model was used to assess the risk of occurrence and predict the strength and timing of a second COVID-19 wave under varying levels of daily contact rate from July 04, 2020. Specifically, the daily contact rate required to suppress the epidemic and prevent a resurgence of COVID-19 cases, and the daily contact rate required to stay within the acute bed capacity of the NEL system without any additional intervention measures after July 2020, were determined across the nine different scenarios. Our results caution against a full relaxing of the lockdown later in 2020, predicting that a return to pre-COVID-19 levels of social contact from July 04, 2020, would induce a second wave up to eight times the original wave. With different levels of ongoing social distancing, future resurgence can be avoided, or the strength of the resurgence can be mitigated. Keeping the daily contact rate lower than 5 or 6, depending on scenarios, can prevent an increase in the number of COVID-19 cases, could keep the effective reproduction number R_e_ below 1 and a secondary COVID-19 wave may be avoided in NEL. A daily contact rate between 6 and 7, across scenarios, is likely to increase R_e_ above 1 and result in a secondary COVID-19 wave with significantly increased COVID-19 cases and associated deaths, but with demand for hospital-based care remaining within the bed capacity of the NEL health and care system. In contrast, an increase in daily contact rate above 8 to 9, depending on scenarios, will likely exceed the acute bed capacity in NEL and may potentially require additional lockdowns. This scenario is associated with significantly increased COVID-19 cases and deaths, and acute COVID-19 care demand is likely to require significant scaling down of the usual operation of the health and care system and should be avoided. Our findings suggest that to avoid future COVID-19 waves and to stay within the acute bed capacity of the NEL health and care system, maintaining social distancing in NEL is advised with a view to limiting the average number of social interactions in the population. Increasing the level of social interaction beyond the limits described in this work could result in future COVID-19 waves that will likely exceed the acute bed capacity in the system, and depending on the strength of the resurgence may require additional lockdown measures.

## Introduction

The world remains in the grip of the COVID-19 pandemic caused by the Severe Acute Respiratory Syndrome-Coronavirus 2 (SARS-CoV-2). Transmission of SARS-CoV-2 is thought to occur primarily via the transfer of viral droplets during close contact between individuals, although fomites and aerosol infections have been reported^1^. As of August 07, 2020, and at the time of writing this paper, over 18.8 million cases and over 708,000 deaths have been reported worldwide^2^. In the UK, since the first two cases were reported on January 31, 2020, and the first reported COVID-19 related death occurred on March 06, 2020, over 300,000 cases and almost 50,000 COVID-19 related deaths have been reported as of August 07, 2020^3^.

The impact of COVID-19 on boroughs in North East London (NEL) (population size 2.1 million)^4^ has been significantly higher than other parts of England and Wales. During the first wave of the epidemic, the boroughs of Newham (144) and Hackney (127) had the highest and third-highest age-standardised mortality rate per 100,000 people in England and Wales^5^, while Tower Hamlets (123), Waltham Forest (93) and Barking and Dagenham (89) also had death rates higher than both the averages for local authority areas across London (86) and England and Wales (36).

The first case of COVID-19 in the region was reported on February 19, 2020, and the first COVID-19 associated death was documented on March 06, 2020^3,6^. Since then the local epidemic has spread rapidly with 7205 confirmed cases (up to August 03, 2020) and 1732 deaths (registered by August 01, 2020) associated with COVID-19 across NEL^3,6^.

**Figure 1:**
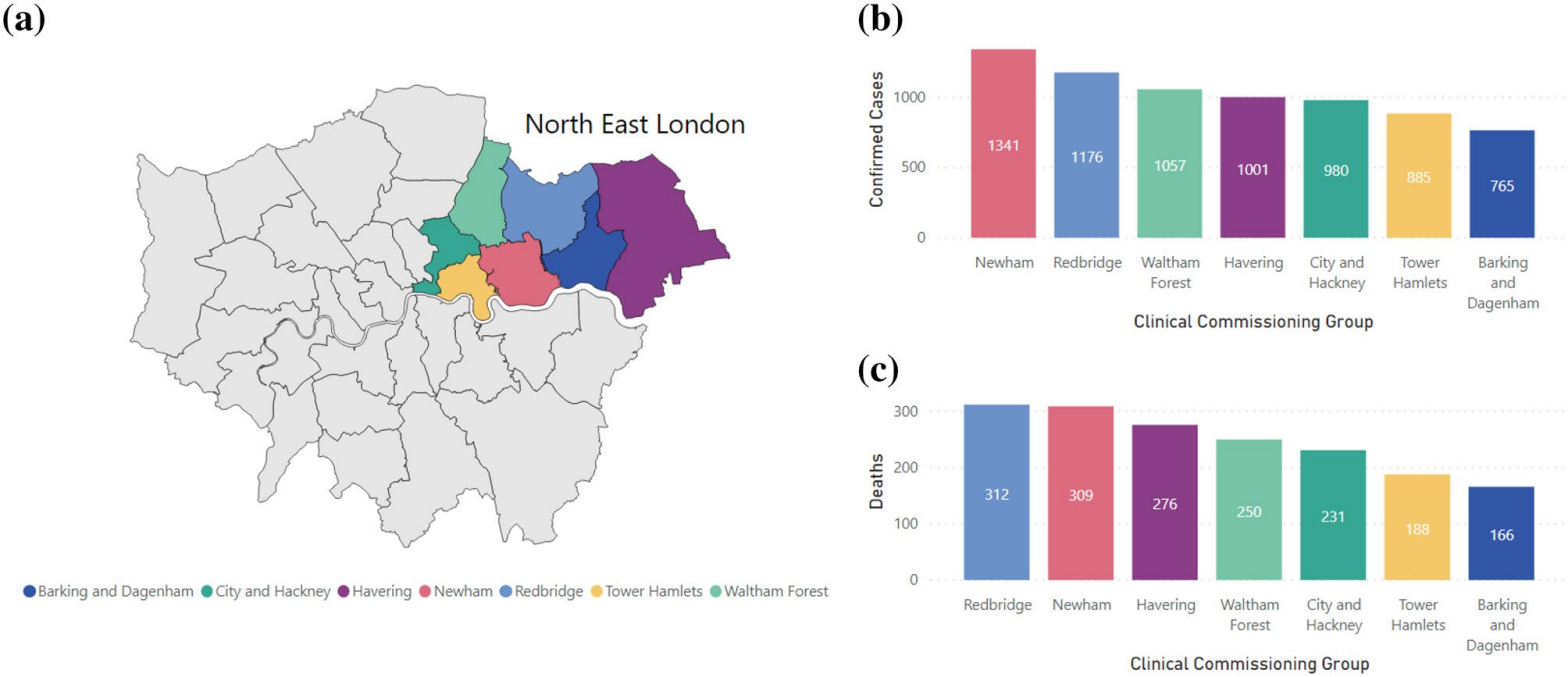
(**a**) NHS Clinical Commissioning Group (CCG) London boundaries, with North East London CCGs highlighted. (**b**) Number of confirmed cases of COVID-19 for North East London Clinical Commissioning Groups, up to 3rd August 2020^3^. (**c**) Number of deaths due to COVID-19 for North East London Clinical Commissioning Groups, for deaths that occurred up to 24th July, registered up to 1st August^4^.

The NEL healthcare system comprises 3 ‘integrated care partnerships’ (ICPs), covering 7 constituent London boroughs: (1) City of London and Hackney (combined in line with commissioning of health and care services by City and Hackney Clinical Commissioning Group (CCG) and referred to throughout as City and Hackney), (2) WEL: Waltham Forest; Newham; Tower Hamlets, (3) BHR: Barking and Dagenham; Havering; and Redbridge. The population of each ICP is served principally by a different acute NHS trust, albeit with some overlap with other areas: City & Hackney is served by Homerton University Hospital NHS Foundation Trust (HUT); WEL is served by Barts Health NHS Trust (Barts); BHR is served by Barking, Havering and Redbridge University Hospitals NHS Trust (BHRUT). Since the onset of the epidemic, data has been collated from these subsystems within NEL^7^ on the number of COVID-19 cases, deaths and hospitalised patients as shown in Fig. 1.

NEL is highly diverse in terms of ethnicity and age as well as socio-economic mix, with large Black, Asian and minority ethnic (BAME) populations with the majority of the population in Newham, Tower Hamlets and Redbridge from BAME groups^4,8^. Furthermore, Barking and Dagenham has the lowest life expectancies for males in London, while Havering has the largest proportion of people aged over 65 years of any London borough^9^. It has been widely reported that these groups, South Asian, Black, over 70s and males, have a significantly higher risk of being hospitalised due to COVID-19^10,11^. Hence, understanding the pressure of COVID-19 on the local health and care system, and the constraints needed to keep infection rates within the hospital capacity levels, is of increased importance in NEL due to its particularly vulnerable population. It is also important to support system planning over the coming 12 months. There is a need to support both COVID-19 care and those with non-COVID-19 health concerns (e.g. type 2 diabetes, cardiovascular disease and cancer, etc.), and consider how much health and care system capacity is available for those with COVID-19 and non-COVID-19 needs.

On March 23, 2020, the UK Government imposed strict social distancing measures (‘lockdown’), to protect the public, slow down the virus spread, and reduce the associated morbidity and mortality and prevent excess demand on the National Health Service (NHS). As the number of daily cases and confirmed deaths started to decline in late April 2020, the first steps were taken towards lockdown easing with the partial reopening of primary schools on June 01, 2020, of secondary schools from June 15, 2020, and non-essential businesses from July 04, 2020. Given the relatively high levels of ongoing infection^3^, it is important to assess whether the number of infections will increase again when lockdown measures are lifted, potentially leading to future epidemic waves and increasing the effective reproduction number R_e_. To suppress the virus and control the epidemic, R_e_ needs to remain below 1. The biggest step in lockdown easing came into effect in England from July 04, when restaurants, pubs and hairdressers reopened, followed by the allowance of two households to meet indoors (where the risk of transmission/survival of the virus is significantly higher) and relaxation of the two-metre social distancing to rule of “one-metre plus”^12^. Lockdown easing increases the daily contact rate and it is critically important to understand the extent to which such increase in the contact rate among people will affect control of COVID-19 transmission or lead to a secondary wave.

Since the onset of the epidemic in the UK, mathematical modelling has been widely used to understand the spread of COVID-19 across different settings^13^, and to design optimal strategies to reduce the future burden and prevent a second wave. Different mathematical models have been utilised: deterministic models on whole populations and rooted in using equations tracking Susceptible-Exposed-Infectious-Removed (SEIR) populations^14^, sometimes age-stratified^15,16^; and stochastic i.e. individual-based models^17,18^ for transmission between individuals in a population. Contact rate, as the daily number of contacts per person per day, is one of the key parameters within each group of models and physical/social distancing measures can be modelled by varying the contact rate. A survey on adults’ behaviour in the UK during a period of lockdown and comparing the results to previously collected data from Polymod^19^, suggest a large reduction in daily contacts particularly outside the home, resulting in a marked reduction in the estimated effective reproduction number from 2.6 to 0.62^15^. In these studies, a contact is defined as encounters with either skin-to-skin contact or a two-way conversation of at least a few words. As the UK uses a phased lockdown release plan, with reopening of schools as the first step of reopening society from September 2020^17^, it is imperative to assess how an increase in contact rate will affect R_e_ and hence ability to suppress the virus in the future. We note that these results depend on the level of compliance with additional countermeasures, including face coverings usage, hand-washing, and correct social distancing.

In this paper, we present an SEIR model for COVID-19 spread across the NEL boroughs of Hackney, Tower Hamlets, Newham, Waltham Forest, Barking & Dagenham, Havering, Redbridge, and the City of London. Within the removed (R) compartment we include multiple sub-compartments to project the following outcomes: daily overall hospitalised patients, daily hospitalised patients within Intensive Care Units (ICUs), daily discharges from hospitals and daily deaths within hospitals from COVID-19. The novelty of our work is in combining the model with granular local data to calibrate these four projections against, and hence generate estimates for the epidemic in NEL.

We aim to determine the level of social distancing compliance, quantified here in terms of the daily number of social contacts per person, needed to maintain future control of the COVID-19 epidemic in North East London and not exceed healthcare capacity in case of secondary waves, via a series of modelling simulations.

## Methods

### Mathematical model

**Figure 2:**
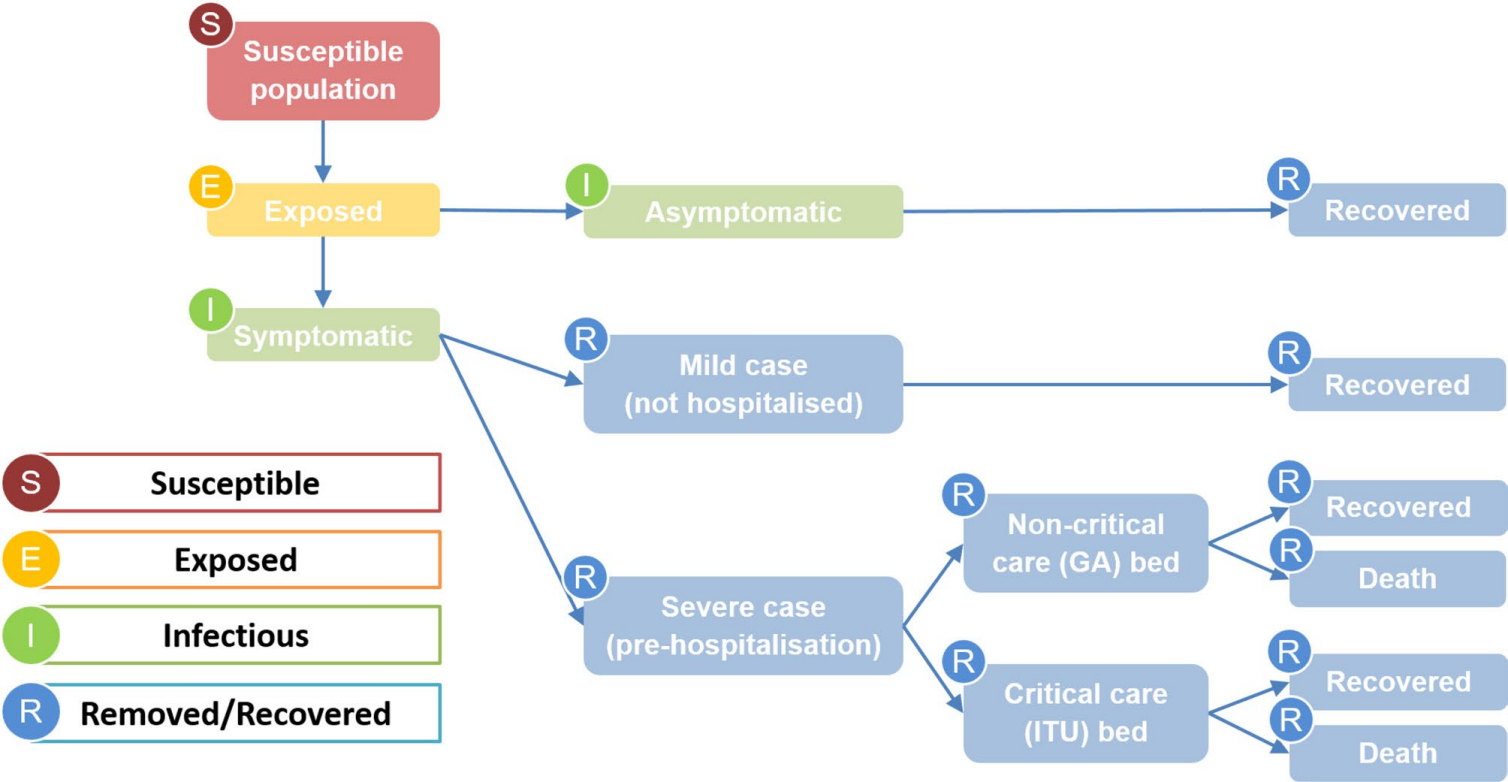
Schematic of the possible pathways within the SEIR epidemiological model. Arrows show the progression through the stages from susceptible to removed/recovered.

We developed a Susceptible-Exposed-Infectious-Removed (SEIR) mathematical model to simulate the spread of COVID-19 in NEL as shown in Fig. 2. Details of the model equations, parametrisation and calibration of the model are contained in the supplementary material.

We assume that SARS-CoV-2 is introduced into a susceptible population of around 2.1 million people, resembling the population of NEL, on 16th February 2020. Those exposed to the virus become infectious after a median incubation period of 5.1 days^20^.

Within the model, we account for the proportions of people infected that have symptoms *(symptomatic)* vs those that do not show symptoms (*asymptomatic*) and we introduce a parameter that quantifies the proportion of symptomatic cases out of all cases (*ρ*_*sym*_ with details in the supplementary material). In the literature, there is mixed evidence on what this proportion is^21^. Hence we explored three values for this parameter, assuming 70% for the main study and varying it to 50% and 25% in the sensitivity analysis (details in the supplementary material).

Additionally, we also vary the infectiousness period within the model. Firstly, in absence of data to suggest otherwise, the infectious period is treated as equal for both symptomatic and asymptomatic cases and as a population average of all cases. A recent literature review suggested that COVID-19 infectiousness peaks in the first 5 days following symptom onset, while no live virus has been cultured after 9 days following symptom onset^22^. Based on this, and taking in consideration that the single population average parameter for infectiousness period is likely to be shortened by self-isolation of a significant proportion of symptomatic patients, we simulated three scenarios for the infectiousness periods of 1, 3 and 5 days.

In our modelling approach, we divide the removed compartment into multiple sub-compartments to reflect differential pathways for patients depending on the severity and outcome of infection. Cases are divided into asymptomatic, mild (not hospitalised) and severe (hospitalised), based on the proportion of symptomatic cases, *ρ*_*sym*_, and the proportion of symptomatic cases requiring hospitalisation, *ρ*_*severe*_. This is detailed in the supplementary material.

Hospitalised cases are divided into critical and non-critical, with the former requiring an ICU bed, based on the proportion of patients requiring critical care, *ρ*_*critical*_. For each of these two hospital pathways, there are distinct parameters associated with patient mortality rate and length of stay in the hospital, based on observed differences^23^. The model focuses on hospitalised cases only for healthcare planning, not taking account of any out-of-hospital deaths occurred. Further details can be found in the supplementary material.

### Data

The number of daily patients hospitalised with COVID-19, in both non-critical and critical care beds, and the number of daily discharges of COVID-19 patients was obtained from the COVID-19 Dashboard produced by NHS England and NHS Improvement^24^. The number of daily COVID-19 hospital deaths were provided by NHS England^25^.

All data was provided at a trust level. Data was aggregated to include the relevant NHS Trusts providing acute care in North East London: Barking, Havering and Redbridge University Hospitals NHS Trust; Barts Health NHS Trust; and Homerton University Hospital NHS Foundation Trust.

Estimates of hospital capacity were derived from local COVID-19 response plans and reported bed capacity data during the COVID-19 pandemic^24,26^. This reflects considerable work done to increase overall hospital bed capacity within NEL, and London. Information on capacity includes general acute beds as well as critical care capacity, it notes current capacity as well as additional planned capacity. Capacity is broken down into 3 levels: (i) currently available; (ii) planned capacity; and (iii) planned capacity plus Nightingale hospital bed capacity. Estimates account for infection control planning to maintain certain hospital sites as COVID-free zones (equating to approximately 20–25% of overall and critical care bed capacity) to maintain a degree of non-COVID-19 care. Capacity estimates are used to assess where a secondary epidemic wave is likely to exceed acute bed capacity of the NEL health and care system.

### Parametrisation and calibration

The model is parametrised with a combination of fixed and fitted parameters listed in Tables S2 and S3. The model was calibrated against observed data for NEL on COVID-19 hospitalised patients (both non-critical and critical care), discharges and in-hospital deaths, including all data up to July 01, 2020^24,25^. One of the key fitted parameters in the calibration was the daily contact rate which was allowed to vary as a step function following key policy decisions such as lockdown, as well as periodically during lockdown measures, to allow the force of infection to modulate over time. Details with fitted parameters used to calibrate the model are listed in Table S3. Further details can be found in the supplementary material.

### Analysis

For the main analysis of this paper, we varied the daily contact rate, *c*, between 3 (approximating the measured value during lockdown of 3.1) and 12 (slightly higher than the pre-COVID-19 UK average of 10.7), from July 04, 2020, within the calibrated model. The daily contact rate is held constant from this date forwards, after being modulated as a fitted parameter during calibration with a series of step-functions over time. We projected the number of daily COVID-19 cases, associated deaths and overall and ICU hospitalised patients. We used the model projections to derive the maximum *c*, across different scenarios, to avoid a second COVID-19 wave, keep R_e_ below 1 and not exceed the acute bed capacity in NEL.

Since there is uncertainty about the length of time for which people are infectious, and the proportion of people with symptomatic infection, we varied the parameter that describes the infectiousness period, and the proportion of symptomatic cases, *ρ*_*sym*_. The infectiousness period was set to 5 days and the proportion of symptomatic cases set to be 70% for the main analysis, based on published work^15,17^. We note that a single value is used to represent the infectiousness period and the contact rate for all individuals, for both symptomatic and asymptomatic infection. In reality, we may expect these values to differ for symptomatic and asymptomatic cases due to possible differences in infectivity profiles as well as social behaviour. But in absence of reliable estimations for these differences, since asymptomatic infection is difficult to measure, we use a single value for these parameter to avoid adding further uncertainty into the model.

### Ethics approval and consent to participate

This study used secondary anonymised data set for which no ethics approval or consent to participate was required.

## Results

Across all nine scenarios, for varying proportion of symptomatic cases and infectiousness period, the model was calibrated to closely match the NEL observed data (Figure S1 in the supplementary material). All fitted parameters are summarised in Table S3 of the supplementary material.

**Figure 3:**
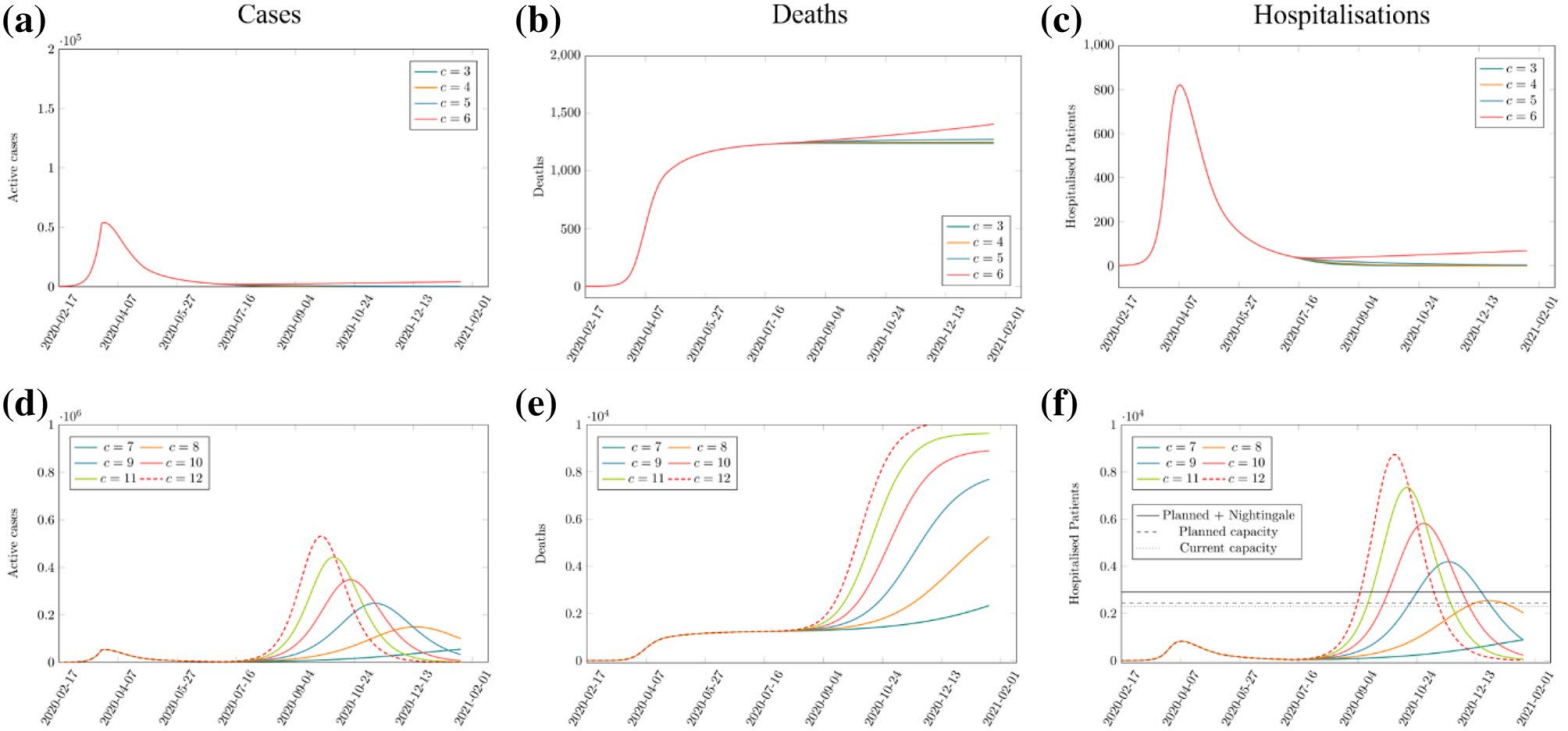
Projections of the mathematical model forecasting the number of COVID-19 cases (left panels), deaths (centre panels) and hospitalised patients (right panels) for varying numbers of daily contacts, *c*. (**a**)–(**c**) show high levels of social distancing, *c* = 3 to 6, and (**d**)–(**f**) show low social distancing, *c* = 7 to 12. Infectiousness period = 5 days, proportion of symptomatic infections = 70%. Estimates for hospital capacity levels are given in (**f**) for reference.

Figure 3 shows the model-projected daily cases, cumulative deaths and daily number of hospitalised patients for varying levels of daily contacts per person (or daily contact rate), *c*, for a population-average infectiousness period of 5 days and a proportion of symptomatic infection of 70%. Our results suggest that significant relaxation of social distancing measures in NEL, with an average of more than 6 daily contacts per person from 4th July 2020 leads to a resurgence of COVID-19 cases and a secondary epidemic wave (Fig. 3d–f). The size of a secondary COVID-19 wave depends on the level of social distancing compliance, i.e. on the average number of daily contacts per person.

**Figure 4:**
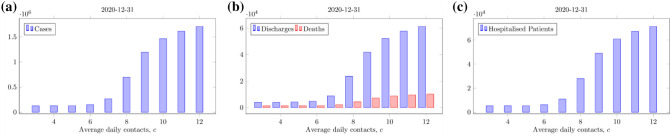
Cumulative number of COVID-19 cases (**a**), hospital discharges and deaths associated with COVID-19 (**b**) and hospitalised patients with COVID-19 (**c**) for different levels of compliance with social distancing quantified by the number of daily contacts, *c*, from 4 July 2020. The cumulative totals for 2020 are considered, including forecasts up to 31 December 2020. For *c* > 6 the number of cases, deaths, hospitalised patients and discharges increase significantly. The results shown here assume an infectiousness period of 5 days and that symptomatic infections comprise 70% of all infections.

With full relaxation of social distancing and return to pre-COVID-19 levels of social contact (*c* ≈ 11), a secondary COVID-19 wave may occur up to 8 times larger than the original wave in terms of number of infections (Fig. 3d), peak number of patients hospitalised with COVID-19 and associated deaths (Figs. 3f, 4b). In addition to a surge in the number of daily cases, hospitalised patients and deaths, the health and care demand will exceed the acute bed capacity in NEL in this scenario.

**Figure 5:**
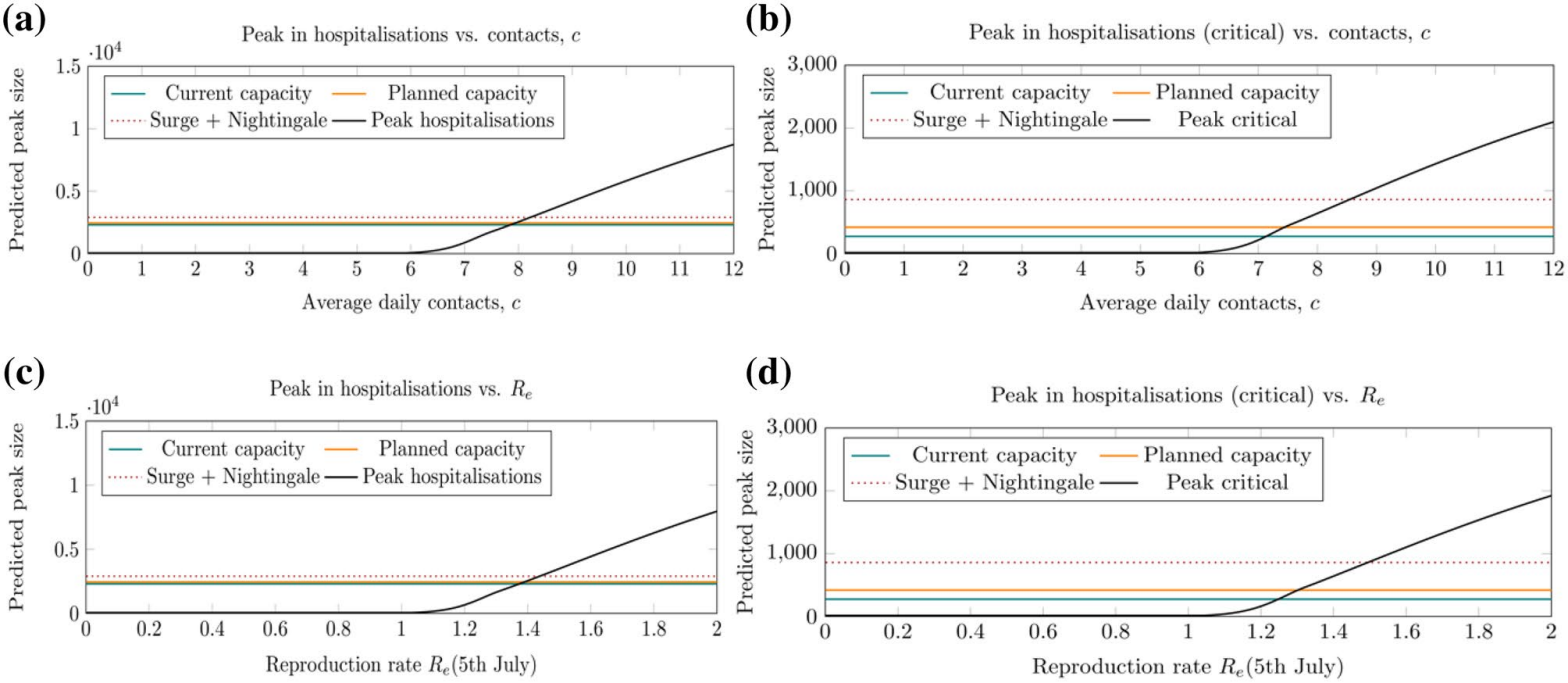
Predicted size of the secondary COVID-19 wave (black lines), measured by the peak in all COVID-19 hospitalised patients (left panels) and critical care COVID-19 hospitalised patients (right panels) for different levels of compliance with social distancing from 4 July 2020 as a function of the daily number of contacts, *c*, (top row) and the effective reproduction number, R_e_ (on 5 July 2020, bottom row). Estimates for hospital capacity levels (coloured lines) are given for reference. For *c* > 6, R_e_ is above 1 and a secondary wave is predicted. The results shown here assume an infectiousness period of 5 days and that symptomatic infections comprise 70% of all infections.

At the time of submission, analysis suggested that a secondary COVID-19 wave, including excess demand on acute care, may be prevented when a sufficient level of social distancing remains in place. Specifically, to prevent a significant secondary wave in NEL, the average daily contact rate after July 04 must not exceed 6 (Fig. 3a–c). With this level of compliance with social distancing, the burden from COVID-19 would be less in terms of total cases, hospitalised patients and deaths (Fig. 4a–c), and the acute bed capacity demand not exceeded (Fig. 5a,b) and R_e_ will remain below 1 (Fig. 5c,d). Hence avoiding a secondary wave of COVID-19 in NEL would require reduction in pre-COVID-19 average daily contact rate to around 50% of its pre COVID-19 level in the region indefinitely in the near term. While our analysis examined the period up to early 2021 only, the principle of our analysis remain the same for looking further forwards in time—future epidemic waves require R_e_ < 1 for suppression, which in our results corresponds to an average daily contact rate of up to 6.

A secondary wave remains within the bed capacity of the health and care system for an average number of daily contacts per person of up to 7 to 8 (Fig. 5a,b). However, this scenario is associated with a significantly increased numbers of total cases, hospitalised patients, discharges and deaths (Fig. 5). A daily contact rate of 8 gives an end-of-year death total of over 4000, a more than threefold increase compared to if daily contacts were kept to 6 or lower. Going from a population average rate of daily contacts of 8 to 9 increases the peak in COVID-19 hospitalised patients from 2500 to 4200, crossing all 3 overall bed capacity scenarios, including the maximum capacity (current plus planned plus Nightingale) of approximately 2900 beds (Fig. 3).

When we vary the infectiousness period to be 3 or 1 days, and the proportion of symptomatic infection to be 50% or 25%, the overall results remain consistent, with a secondary epidemic wave present unless restricted social distancing is present. Across all 9 scenarios, for varying levels of infectiousness period and proportion of symptomatic infection, the limit on the average number of daily contacts to suppress a secondary wave is between 5 and 6, while the maximum average number of contacts for a secondary peak to remain within NEL capacity levels is between 8 (in 7 scenarios) and 9 (in 2 scenarios). Across scenarios, a longer infectiousness period pushes a secondary wave further into the future, and a lower proportion of symptomatic infections leads to a smaller peak in hospitalised patients for equivalent *c* values. Therefore, we find it is the balance between infectiousness period and the proportion of infections that are symptomatic that controls the timing and the strength of a potential secondary wave in NEL. Results for all 9 scenarios are summarised in Figures S1–S12 of the supplementary material.

## Discussion

Our findings suggest that a limited relaxation of the level of social distancing in NEL is necessary to avoid future COVID-19 waves and not exceed the acute bed capacity in the region. Good compliance with social distancing measures and a maximum of 5–6 daily contacts per person on average, equivalent to a 45–55% reduction in pre-COVID-19 average daily contacts, is necessary to keep the virus suppressed and keep the effective reproduction number R_e_ below 1, across all nine scenarios modelled, varying the infectiousness period and proportion of symptomatic infections.

Our results suggest that lockdown is a highly effective strategy in reducing infections and mortality, and that lifting of lockdown fully in the near term, prior to mass vaccination of the population, would likely lead to a resurgence of cases and a secondary COVID-19 peak, as shown by other modelling studies^14–18^.

We find that if the average daily contact rates per person increase to 7–8, while a secondary COVID-19 wave will occur, the increased number of hospitalised patients will remain within the acute bed capacity of the local health and care system. However, such scenarios are highly undesirable, leading to multi-fold increases in the numbers of cases, hospitalised patients, discharges and deaths. As seen during the primary epidemic wave, a surge in COVID-19 hospitalised patients can lead to severe disruption of the health and care system leaving patients unable to receive the care they require, with elective procedures postponed or cancelled, and likely avoidance of attendance due to fears around infection leading to an increase in excess deaths^27^. Furthermore, the infection risk to staff of having a significant proportion of hospital capacity used by COVID-19 patients places immense burden on the local health and care system. Hence, although we find that such scenarios of increased social contact are within certain operational limits, they have many negative consequences and should be avoided.

Our analysis shows the sensitivity of the epidemic to the degree of social contact, with a relatively small increase in average social contact leading to very much worse outcomes in terms of public protection and stress to the health and care system. We note here, as discussed in more detail below, that our model does not account for the varying level of risk of COVID-19 on different segments of the population, whose ability to safely engage in social contact will vary, e.g. comparison of lower-risk population such as children vs. older generations. Additionally, the relatively broad definition of a contact does not capture the significant differences in the probability of a contact spreading infection, depending on factors such as household composition, location and contact duration. While there is insufficient data on the contact duration necessary for infection to spread, the Centre for Disease Control use an operational definition of more than 15 min in their guidance on exposure to COVID-19^28^. It is unknown what proportion of contacts recorded in literature surveys meet this narrower criteria; however, the number of these ‘higher-risk’ contacts is likely to be lower than the overall number of daily contacts. Our population average social contact parameter, *c*, does not attempt to capture these levels of nuance. Nevertheless, this work further highlights the need for careful and strategic relaxing of social distancing in order to control future COVID-19 outbreaks.

The pertinent question in the UK is when, how, and how much, can social distancing measures be modified while keeping infection under control (R_e_ < 1). Our findings suggest that, if some level of social distancing remains in place, with daily contacts at most 45–55% of that pre-COVID-19 depending on the assumed level of infection that is asymptomatic, a potential second wave would be much less severe. Although previous studies have modelled the potential impact of social distancing on viral spread^15–17^, our study is the first to quantify the average number of daily contacts per person required for viral prevention and control in a setting in the UK. We illustrate that small degrees of relaxation to social distancing measures would allow hospitals and ICUs to operate within capacity. However, any increase in daily contacts above an average of 6 is likely to induce a second wave and over 8 daily contacts on average would exceed the capacity of acute care.

Ongoing surveys on a representative sample of UK adults about their contact patterns on the previous day, by colleagues at London School of Hygiene and Tropical Medicine (LSHTM) suggest that the number of daily contacts reduced 73% with the imposed lockdown measures from March 2020^15^. More recent data reveal that although there was a small increase in the average number of contacts per person per day in the first 2 weeks of July, with the reopening of non-essential business from July 04, 2020, to around 4 contacts per person per day^15^, this still remains below the limit of 6 that we suggest as necessary to avoid a secondary wave. Hence people remain cautious as society reopens and our findings suggests this needs to remain in place in future.

The measures suggested by our findings to control the spread of COVID-19 later in 2020 and prevent the NEL health care system from exceeding acute bed capacity are in line with the ‘rule of six’ UK policy adopted in September 2020 in the face of increasing cases^29^. These measures are avoiding large gatherings, and avoiding close contact with more than six people per day. The introduction of phased relaxation of lockdown measures, such as reopening of schools and businesses, are important to protect education and the economy. However, while infections are still present, it is important to keep tracking the epidemic trends and react in a timely way to any future surge, as has been the case in other countries^30^. In particular, close attention should be paid to monitoring population groups with unique risks such as those living in intergenerational and overcrowded households, where people with a lower risk of COVID-19 complications mix with those who have greater COVID-19 risk levels.

While lockdown strategies can suppress the virus, reopening of society is important to protect the economy. Maintaining the balance between saving lives and saving livelihoods is crucial^31^. It is also important to allow people to maintain important social links more easily, particularly where individuals are reliant on public transport and other more shared spaces to do so. Phased reopening of society, with preparedness to react quickly if the epidemic metrics start to surge are crucial as we plan for future waves. Our results give quantification on the level of compliance with social distancing that is necessary to prevent future secondary waves.

The work we present here has some limitations. Our model does not include granular population structure, due to limitations of the observed data. Data available at the smaller geographic areas of interest used as inputs in our model, is available as aggregated totals only for hospitalised patients, hospital discharges and hospital deaths, without any demographic detail such as age, gender or ethnic background. As a result, our model is unable to reliably use an age-stratified model without making assumptions about the observed data. Because of this, we are not able to consider age stratification and the associated risks of different age groups. Due to these limitations of the observed data, we have used a single parameter to describe the average daily contact rate, rather than an age stratified rate or a contact matrix representing mixing within and between population groups. We do not consider contact patterns characteristic of population groups with different employment types, household compositions or social behaviours. Hence the amount of social contacts per person per day derived here as necessary for future outbreak control is an average value for the entire population. This means our result of 5–9 contacts as the limit for our system is not a hard line for all individuals and will vary depending on individual COVID-19 risk. For example, with reopening of schools, school-age children returning to school may have more than 8 contacts per day while having lower risk of COVID-19, while older generations are at much higher risk and so may remain shielded with contacts well below 8. We also note that neglecting population structure can overestimate the size of outbreaks^32^. This is due to the susceptible subpopulations of different groups actively shielding or becoming depleted. Furthermore, the broad definition of a contact means that all contacts are not equal in terms of probability of spreading infection. As a result, we expect our study to underestimate the average contact rate that is safe for avoiding a second wave.

While we have made every effort to characterise the pandemic in a way that resembles that of the UK, some of the parameters we have used are from a variety of sources across different settings within the published literature. Since the purpose of the study was a large scale sensitivity analysis on the impact of the daily contact rate on mitigating a secondary pandemic wave, such parametrisation is sufficient. We did not consider other control measures that might decrease the effective reproduction number, e.g. shielding of vulnerable population groups. While relaxing these assumptions may affect outcomes such as the size of a secondary peak quantitatively, we don’t expect these to change the overall message of our work.

We note that our study only looks at contact rate as the main driver of transmission of the virus and does not account for behavioural changes that alter transmission in some other way. For example, if people follow guidance on social distancing and mask wearing even when socially mixing, this may reduce the transmission probability even as a given level of contact is maintained^33^. We have not considered the policy of compulsory face coverings imposed in England from 24th July 2020. But we note that if we assume 45% mask efficacy with 70% compliance, resulting in a 30% overall reduction in risk, then the number of effective contacts could be 30% higher than the values reported here. Incorporating this and other non-pharmaceutical interventions within the model is something we plan to explore in future. Additional, complementary work on this is ongoing by some of the authors, focusing on assessing the impact of this policy combined with reopening schools and society from September 2020 and with ongoing test-trace-isolate strategy on the COVID-19 epidemic.

The methodology we have used here can easily translate to other settings. NHS Right Care^27^ have a tool that uses demographic factors (deprivation, age, population and ethnicity) to compare different Clinical Commissioning Groups (CCGs) and provides a ‘nearest-10’ comparator group for each CCG. By identifying the CCGs that are similar to those in NEL, future work can compare COVID-19 deaths and hospitalised patients across such CCGs and use these to re-calibrate the model. This will allow us to explore whether the compliance with social distancing that we are suggesting here is applicable to similar CCGs.

In summary, we show that limited relaxing of the lockdown in NEL is necessary to avoid secondary COVID-19 waves and not exceed the acute bed capacity in the health and care system. Good compliance with social distancing measures, with a maximum of 5–6 daily contacts per person on average (i.e. a 45–55% reduction in pre-COVID-19 average daily contacts), is necessary to keep COVID-19 suppressed and keep R_e_ below 1.

## Supplementary Information

Supplementary Information.

## Data Availability

The datasets used and analysed during this study and the numerical codes used to generate the outcomes of this paper are available from the corresponding author on reasonable request.
