# Supplementary material for: Determining the level of social distancing necessary to avoid future COVID-19 epidemic waves: a modelling study for North East London
Source: Sci Rep. 2021 Mar 11;11:5806. doi: 10.1038/s41598-021-84907-1 (PMC7952900; doi:10.1038/s41598-021-84907-1)
Supplement: Supplementary file 1 — Supplementary Information. [file 41598_2021_84907_MOESM1_ESM.pdf]

Supplementary material to
Determining the level of social distancing necessary to avoid future COVID-19 epidemic waves: a modelling study for North East London

Nathan Cheetham[1,*], William Waites[2], Irene Ebyarimpa[1], Werner Leber[3], Katie Brennan[1] and Jasmina Panovska-Griffiths[4,5,6*&]

[1] Financial Strategy Team, NHS North East London Commissioning Alliance, London, UK
[2] University of Edinburgh, Edinburgh, UK
[3] Centre for Clinical Effectiveness and Health Data Science, Institute of Population Health Sciences, Barts School of Medicine and Dentistry, Queen Mary University of London, London, UK
[4] Department of Applied Health Care, Institute of Epidemiology & Health Care, University College London, London
[5] Institute for Global Health, University College London, London, UK
[6] The Queen's College, University of Oxford, Oxford, UK

*=contributed equally
&=corresponding author: j.panovska-griffiths@ucl.ac.uk

Mathematical model

Our model is a classic SEIR model where the system of equations (1)-(17) models the transmission of the coronavirus - SARS-CoV-19 virus- among a population of people susceptible to the virus (S), those exposed to the virus (E), those infectious with the virus (I) and those removed from the model (R). The schematic of the model is shown in Figure 2 of the main manuscript. Here we describe the specific equations, different sub cohorts of I and R and the terms within the equations.

The infectious compartment is divided into symptomatic, I_{sym}, and asymptomatic, I_{asym} infections, with a parameter that quantifies the proportion of all cases that are symptomatic cases defined as ρ_{sym}. In the literature, there is a mixed evidence on what this proportion is [1]. Hence we explored three values for this parameter ρ_{sym}, assuming ρ_{sym}=70% for the main study and varying it to 50% and 25% in the sensitivity analysis.

People exposed to the virus become infectious with a force of infection $\beta c(I_{sym} + I_{asym})$ where β represents the probability of transmission, and is related to their viral load, and c is the average number of contacts per person per day. Following the conventions of previous literature [2], and in the context of COVID-19 transmission, a contact is defined as either an encounter with another person involving skin-to-skin contact, or a two-

way conversation without physical contact. Importantly, the effective transmission rate, βc, is modelled as a step function, varying at the times listed below.

$$\beta c = \begin{cases} \beta_0 c_0, t < 16\ March\ 2020 \\ \beta_1 c_0, t \in (16\ March\ 2020, 23\ March\ 2020) \\ \beta_2 c_1, t \in (23\ March\ 2020, 29\ April\ 2020) \\ \beta_2 c_2, t \in (29\ April\ 2020, 28\ May\ 2020) \\ \beta_2 c_3, t \in (28\ May\ 2020, 4\ July\ 2020) \\ \beta_2 c_4, t \in (4\ July\ 2020\ onwards) \end{cases}$$

The initial effective transmission rate, $\beta_0 c_0$, is derived from a fitted basic reproduction number, R_0, and a rate of contacts, c_0, of 10.7, is based on previous studies of average contact rates in the United Kingdom [4] For each of the time points at which βc can vary, representing changes in government policy and/or population behaviour, either β or c is held constant, and the other allowed to vary as a fitting parameter. The contact rate during the first period of lockdown, c_1, is fixed at 3.1 based on the observed value reported in the literature [5], and subsequently allowed to vary as informed by the phased relaxing of the lockdown measures whilst β remains fixed to the calibrated value.

A recent literature review suggested that COVID-19 infectiousness peaks in the first 5 days following symptom onset, while no live infection has been cultured after over 9 days following symptom onset [3]. Based on this, and taking in consideration that the single population average parameter for infectious period is likely to be shortened by self-isolation of a significant proportion of symptomatic patients, we simulated three scenarios for the infectiousness periods of 1, 3 and 5 days, with infectious period of 5 days used in the main study, and 1 and 3 days in the sensitivity analysis.

$$\frac{dS(t)}{dt} = -\beta c.(I_{sym} + I_{asym}).S(t) \quad (1)$$

$$\frac{dE(t)}{dt} = \beta c.(I_{sym} + I_{asym}).S(t) - \alpha.E(t) \quad (2)$$

$$\frac{dI_{sym}(t)}{dt} = \rho_{sym}.\alpha.E(t) - \gamma_{sym}.I_{sym}(t) \quad (3)$$

$$\frac{dI_{asym}(t)}{dt} = (1 - \rho_{sym}).\alpha.E(t) - \gamma_{asym}.I_{asym}(t) \quad (4)$$

The removed compartment (R) is divided into sub compartments to reflect the various pathways for patients depending on the severity and outcome of the COVID-19 infection. Cases are divided into asymptomatic, mild (not hospitalised) and severe (hospitalised), based on the proportion of symptomatic cases, ρ_{sym}, and the proportion of symptomatic cases requiring hospitalisation, ρ_{severe}.

Asymptomatic and “Mild” symptomatic cases have the following 2-stage routing to recovery following infection:

$$\frac{dR_{asymptomatic}(t)}{dt} = \gamma_{asym}.I_{asym}(t) - \frac{1}{t_{recovery(asymptomatic)}}R_{asymptomatic}(t) \quad (5)$$

$$\frac{dR_{recovered(asymptomatic)}(t)}{dt} = \frac{1}{t_{recovery(asymptomatic)}}R_{asymptomatic}(t) \quad (6)$$

$$\frac{dR_{mild}(t)}{dt} = \rho_{mild}.\gamma_{sym}.I_{sym}(t) - \frac{1}{t_{recovery(mild)}}R_{mild}(t) \quad (7)$$

$$\frac{dR_{recovered(mild)}(t)}{dt} = \frac{1}{t_{recovery(mild)}}R_{mild}(t) \quad (8)$$

Since this work concerns with acute capacity planning, we focus on the acute pathways, and do not account for deaths out of hospital, and the time to recovery/death for asymptomatic and mild symptomatic cases is not directly relevant and set to an arbitrary value of 7 days.

Cases that require hospitalisation pass through multiple stages, first entering the 'pre-hospitalised' population. Cases are split according to whether critical care (ICU/ITU bed) is required, or only non-critical care (General and Acute, "GA" bed) is needed, based on the proportion, ρ_{ITU}. The parameters $t_{hospitalisation(GA)}$ and $t_{hospitalisation(ITU)}$ refer to the time from symptoms to hospitalisation for non-critical and critical cases.

$$\frac{dR_{pre-hosp}(t)}{dt} = \rho_{severe}.\gamma.I_{sym}(t) - \frac{1}{t_{hospitalisation(GA)}}.(1-\rho_{ITU}).R_{pre-hosp}(t) - \frac{1}{t_{hospitalisation(ITU)}}.\rho_{ITU}.R_{pre-hosp}(t) \quad (9)$$

Non-critical (GA) and critical (ITU) cases then enter a hospitalised component, followed by recovery or fatality, based on the proportion of fatal non-critical cases, $\rho_{fatal(GA)}$, and proportion of fatal critical cases, $\rho_{fatal(ITU)}$ and time to recovery and death for both non-critical and critical cases.

$$\frac{dR_{hospitalised(GA)}(t)}{dt} = \frac{1}{t_{hospitalisation(GA)}}.(1-\rho_{ITU}).R_{pre-hosp}(t) - \frac{1}{t_{recovery(GA)}}.\left(1-\rho_{fatal(GA)}\right).R_{hospitalised(GA)}(t) - \frac{1}{t_{fatal(GA)}}.\rho_{fatal(GA)}.R_{hospitalised(GA)}(t) \quad (10)$$

$$\frac{dR_{hospitalised(ITU)}(t)}{dt} = \frac{1}{t_{hospitalisation(ITU)}}.\rho_{ITU}.R_{pre-hosp}(t) - \frac{1}{t_{recovery(ITU)}}.\left(1-\rho_{fatal(ITU)}\right).R_{hospitalised(ITU)}(t) - \frac{1}{t_{fatal(ITU)}}.\rho_{fatal(ITU)}.R_{hospitalised(ITU)}(t) \quad (11)$$

$$\frac{dR_{recovered(severe)}(t)}{dt} = \frac{1}{t_{recovery(GA)}}.\left(1-\rho_{fatal(GA)}\right).R_{hospitalised(GA)}(t) + \frac{1}{t_{recovery(ITU)}}.\left(1-\rho_{fatal(ITU)}\right).R_{hospitalised(ITU)}(t)$$

$$\frac{dR_{fatality}(t)}{dt} = \frac{1}{t_{fatal(GA)}} \cdot \rho_{fatal(GA)} \cdot R_{hospitalised(GA)}(t) + \frac{1}{t_{fatal(ITU)}} \cdot \rho_{fatal(ITU)} \cdot R_{hospitalised(ITU)}(t) \quad (12)$$

where

$$a = \frac{1}{t_{incubation}} \quad (13)$$

$$\gamma = \gamma_{sym} = \gamma_{asym} = \frac{1}{t_{infectious}} \quad (14)$$

Basic reproduction number, $R_0 = \frac{\beta_0 c_0}{\gamma}$ (15)

Effective reproduction number, $R_e(t) = \frac{\beta c S(t)}{N} \int_0^{\infty} e^{-\gamma} \, dt$ (16)

$\rho_{mild} + \rho_{severe} = 1$ (17)

for symptomatic cases

Non-zero initial conditions are as follows:

$$\frac{dS(t=t_0)}{dt} = N - I_0 \qquad \frac{dI_{sym}(t=t_0)}{dt} = I_0$$

Where *N* is the regional population.

The system of equations (1)-(12) are integrated over a series of time points to generate susceptible, exposed, infected and recovered/removed populations over time. A time step of 0.25 days is used for improved fitting of the day 0 of the model, t_0, and then interpolated to 1 day for calculation of residuals with observed data.

Following integration, further compartments are derived for comparison with observed data:

- $R_{hospitalised(GA)}$ and $R_{hospitalised(ITU)}$ are summed to give $R_{hospitalised(all)}$.
- Daily populations of new discharges and deaths are derived from the cumulative totals given by the $R_{recovered(severe)}$ and $R_{fatality}$ compartments.

Data

The collated data used to calibrate the model against are summarised in Table S1.

Table S1. Summary of COVID-19 datasets used to calibrate SEIR model parameters.

Data	Geography	Reporting frequency	Source	Link
Hospital deaths	Trust-level	Daily (figures revised over time)	NHS England COVID-19 Daily Deaths	https://www.england.nhs.uk/statistics/statistical-work-areas/covid-19-daily-deaths/
Number of patients in Hospital(all, non-critical care, and critical care)	Trust-level	Daily (figures subject to revision over time)	NHS COVID-19 Dashboard (access restricted to NHS users)	https://analytics.improvement.nhs.uk/#/views/Covid-19Dashboard/Coverpage?:iid=1<br><br>We note that this dashboard requires registration and approval to access (available to NHS staff). A publically available version of this data is available with a longer time delay through the following link: https://www.england.nhs.uk/statistics/statistical-work-areas/covid-19-hospital-activity/<br>Unfortunately, the data available through this link is, at the time of writing, not backdated to March.
Discharges	Trust-level	Daily (figures subject to revision over time)	NHS COVID-19 Dashboard (access restricted to NHS users)	https://analytics.improvement.nhs.uk/#/views/Covid-19Dashboard/Coverpage?:iid=1
Population estimates	Borough-level	Every 2 years (Mid-2019 estimates used)	Office for National Statistics (ONS) Subnational population projections for England: 2018-based	https://www.ons.gov.uk/peoplepopulationandcommunity/populationandmigration/populationprojections/bulletins/subnationalpopulationprojectionsforengland/2018based

The following notes/caveats on observed data sources are made:

1. NHSE data on hospital deaths has undergone significant revision over time, with revisions in deaths for a given date of death possible, as testing and reporting evolves. Data was correct at time of collection (04 July 2020).
2. Due to the need for rapid collection and dissemination of COVID-19 related data, observed data on hospitalised patients and discharges is not validated and subject to revision. Data is reported to NHS England/Improvement by NHS Trusts, and the quality of reporting may vary between Trusts.

3. The number of hospitalised patients with confirmed COVID-19 in a bed is reported on a daily basis. The true number may be higher due to a proportion of patients who are suspected cases awaiting confirmation with testing. This data is not backdated following confirmation of a suspected case.
4. On dates where Trusts did not report on the number of hospitalised and discharged patients, missing data was interpolated. Similarly, in instances where observed data on the number of hospitalised and discharged patients appeared inaccurate – e.g. outliers with especially high or low values not in line with adjacent days, data points were replaced with interpolated data to reduce fitting being skewed by unreliable data points.

Calibration process

During the model calibration process, several parameters were fixed, shown in Table S2, and several parameters were allowed to vary, as detailed in Table S3, to fit the model epidemic projections to the observed data. Importantly, sets of fitted model parameters were generated for each of the nine scenarios of varying the infectious period (1,3 and 5 days) and proportion of symptomatic proportion (70%, 50% and 25% of all infections), and summarised in Table S3. Initial guesses and fitting limits were based on literature and observed data. As an example, the initial guesses and bounds for the proportion of hospitalised cases requiring critical care parameter were obtained from analysis of the proportion of critical vs. non-critical beds occupied by COVID-19 patients over time as detailed in the observed local data on hospitalised cases. Therefore the initial guess for the parameter controlling the proportion of hospitalised cases requiring critical care was chosen as 20% to represent the average observed value, and the bounds were set at 15% to 35% based on the limits of the variation observed locally.

The best fits were chosen based on the values that minimised the least square difference between the observed data and the model projected outcomes. Vectors containing residuals between observed and modelled populations are concatenated to allow simultaneous minimisation of the four observed datasets. Observed and modelled populations are normalised to the maximum observed value prior to calculation of residuals and concatenation. The concatenated residual vector is minimised using a least squares fitting method. In addition to the effective transmission rate, βc, the only other parameters which are allowed to vary in time are the mortality rates for all hospitalised cases and length of stay in hospital for patients who recover. This was chosen empirically based on observed data showing a slowing in the rate of deaths in comparison to discharges in the second half of the first pandemic wave [7,8]. These observed changes are modelled by allowing the mortality rates for critical and non-critical cases to vary in time by multiplication with a fitted scaling factor. Similarly, the length of stay for recovering cases also varies with time based on a second scaling factor (the data that informed this modelling decision was pseudonymised patient level information on hospital spells provided by NHS Trusts within North East London, however is not publicly available and available for sharing and so is not given an explicit reference). In both cases, the scaling factors are applied linearly over a fixed period of time to model a more gradual change in patient outcomes over time. The time periods over which these parameters vary was determined empirically, based on obtaining the best calibration between observed and modelled populations.

Table S2. Fixed parameters used in SEIR model.

**Parameter**	**Value**	**Source/Rationale**
Incubation time	5.1 days	[6]
Infectious time	1 day,<br>3 days,<br>5 days	Varied to represent multiple scenarios
Number of initial infections, I_0	1	-
Proportion of symptomatic cases, ρ_{sym} (%)	70%, 50%, 25%	Varied to represent multiple scenarios
Intervention 1 date	16th March 2020	Announcement of UK social distancing measures [9]
Intervention 2 date	23rd March 2020	Announcement of UK "Lockdown" measures [9]
Intervention 3 date	29th April 2020	-
Intervention 4 date (for forecasting scenarios)	4th July 2020	Announcement of easing of "Lockdown" measures in England, commencing 4th July [9]
Time from end of infectious period to recovery for asymptomatic cases, $t_{recovery(asymptomatic)}$	7 days	This value is arbitrary, as this compartment does not feed directly into acute demand planning.
Time from end of infectious period to recovery for mild (non-hospitalised) symptomatic cases, $t_{recovery(mild)}$	7 days	This value is arbitrary, as this compartment does not feed directly into acute demand planning.

Table S3. Fitted parameters used in SEIR model, for varying permutations of infectious period, $t_{infectious}$, and proportion of symptomatic cases, ρ_{sym}.

**Parameter**	**Fitted value**								
	**$t_{infectious}$ = 5 days**			**$t_{infectious}$ = 3 days**			**$t_{infectious}$ = 1 day**		
	**ρ_{sym} = 70%**	**ρ_{sym} = 50%**	**ρ_{sym} = 25%**	**ρ_{sym} = 70%**	**ρ_{sym} = 50%**	**ρ_{sym} = 25%**	**ρ_{sym} = 70%**	**ρ_{sym} = 50%**	**ρ_{sym} = 25%**
Basic reproduction number, R_0	3.50	3.38	3.41	3.16	3.19	3.28	3.30	3.30	3.36
βc following Intervention 1, as a proportion of initial *βc* (%)	90%	69%	69%	67%	69%	69%	69%	69%	69%
βc following Intervention 2, as a proportion of initial *βc* (%)	17%	18%	19%	17%	20%	22%	17%	17%	23%
βc following Intervention 3, as a proportion of initial *βc* (%)	22%	23%	24%	31%	30%	30%	31%	31%	29%
Day 0 of SEIR model, t_0	23/01/2020	15/01/2020	13/01/2020	25/01/2020	25/01/2020	25/01/2020	16/02/2020	16/02/2020	16/02/2020
Time from end of incubation to hospitalisation (non-critical care cases), $t_{hospitalisation(GA)}$	3.1 days	4.3 days	3.7 days	7.6 days	6.2 days	4.3 days	8.3 days	8.2 days	4.5 days
Time from end of incubation to hospitalisation (critical care cases), $t_{hospitalisation(ITU)}$	5.1 days	6.2 days	6.7 days	9.2 days	7.6 days	8.9 days	12.5 days	10.6 days	7.6 days
Time from hospitalisation to recovery (non-critical care cases), $t_{recovery(GA)}$	6.3 days	6.1 days	3.1 days	6.2 days	5.8 days	6.7 days	5.6 days	5.6 days	5.8 days
Time from hospitalisation to recovery (critical care cases), $t_{recovery(ITU)}$	11.3 days	9.6 days	10.6 days	11.6 days	10.7 days	11.5 days	10.3 days	11.0 days	10.8 days
Time from hospitalisation to death (non-critical care cases), $t_{fatality(GA)}$	5.1 days	6.4 days	6.4 days	5.0 days	6.5 days	5.0 days	5.4 days	5.5 days	5.6 days
Time from hospitalisation to death (critical care cases), $t_{fatality(ITU)}$	20 days	17.7 days	19.9 days	13.8 days	14.0 days	9.2 days	18.3 days	13.3 days	20.0 days
Proportion of cases requiring hospitalisation, ρ_{severe} (%)	6.0%	4.7%	6.0%	3.1%	3.4%	4.5%	4.5%	6.2%	5.8%
Proportion of hospitalised cases requiring critical care, ρ_{ITU} (%)	21.9%	21.9%	24.6%	18.4%	19.2%	32.1%	20.4%	19.1%	22.1%
Proportion of fatal non-critical care hospitalised cases, $\rho_{fatal(GA)}$ (%)	29.3%	34.3%	34.7%	28.2%	33.8%	23.6%	32.0%	30.1%	33.4%
Proportion of fatal critical care hospitalised cases, $\rho_{fatal(ITU)}$ (%)	60.0%	59.9%	59.8%	55.1%	60.0%	56.1%	60.0%	60.0%	60.0%
Recovery time scaling factor (varied linearly between 22/04/20 and 25/04/20)	1	1.10	1.10	1.12	1.11	1.03	1.32	1.32	1.08

Mortality rate scaling factor (varied linearly between 08/04/20 and 25/04/20)	0.38	0.40	0.40	0.32	0.41	0.36	0.32	0.34	0.35

References

1. Gao, Z. et al. A systematics review of asymptomatic infections with COVID-19. *J. Microbiol. Immunol. Infect.* Epub ahead of print; https://dx.doi.org/10.1016%2Fj.jmii.2020.05.001 (2020).
2. Docherty, A. B. et al. Features of 20133 UK patients in hospital with covid-19 using the ISARIC WHO Clinical Characterisation Protocol: prospective observational cohort study. *BMJ*. 369, 1985; 10.1136/bmj.m1985 (2020).
3. Cevik, M. et al. SARS-CoV-2, SARS-CoV, and MERS-CoV viral load dynamics, duration of viral shedding, and infectiousness: a systematic review and meta-analysis. *The Lancet Microbe.* 10.1016/S2666-5247(20)30172-5 (2020).
4. Mossong, J. et al. Social contacts and mixing patterns relevant to the spread of infectious diseases. *PLoS Med.* **5**, 3; 10. 1371/journal.pmed.0050074 (2008).
5. Jarvis, C. I. et al. Quantifying the impact of physical distance measures on the transmission of COVID-19 in the UK. *BMC Med.* **18**, 124; https://doi.org/10.1186/s12916-020-01597-8 (2020).
6. Stephen, A. et al. The incubation period of coronavirus disease 2019 (COVID-19) from publicly reported confirmed cases: estimation and application. *Ann. Intern. Med.* **172**, 9; 10.7326/M20-0504 (2020).
7. NHS England and NHS Improvement, COVID-19 Dashboard. https://analytics.improvement.nhs.uk/#/views/Covid-19Dashboard/Coverpage?:iid=1 (2020).
8. NHS England. COVID-19 Daily Deaths. https://www.england.nhs.uk/statistics/statistical-work-areas/covid-19-daily-deaths/ (2020).
9. The Health Foundation. COVID-19 policy tracker. https://www.health.org.uk/news-and-comment/charts-and-infographics/covid-19-policy-tracker (2020).

Figures

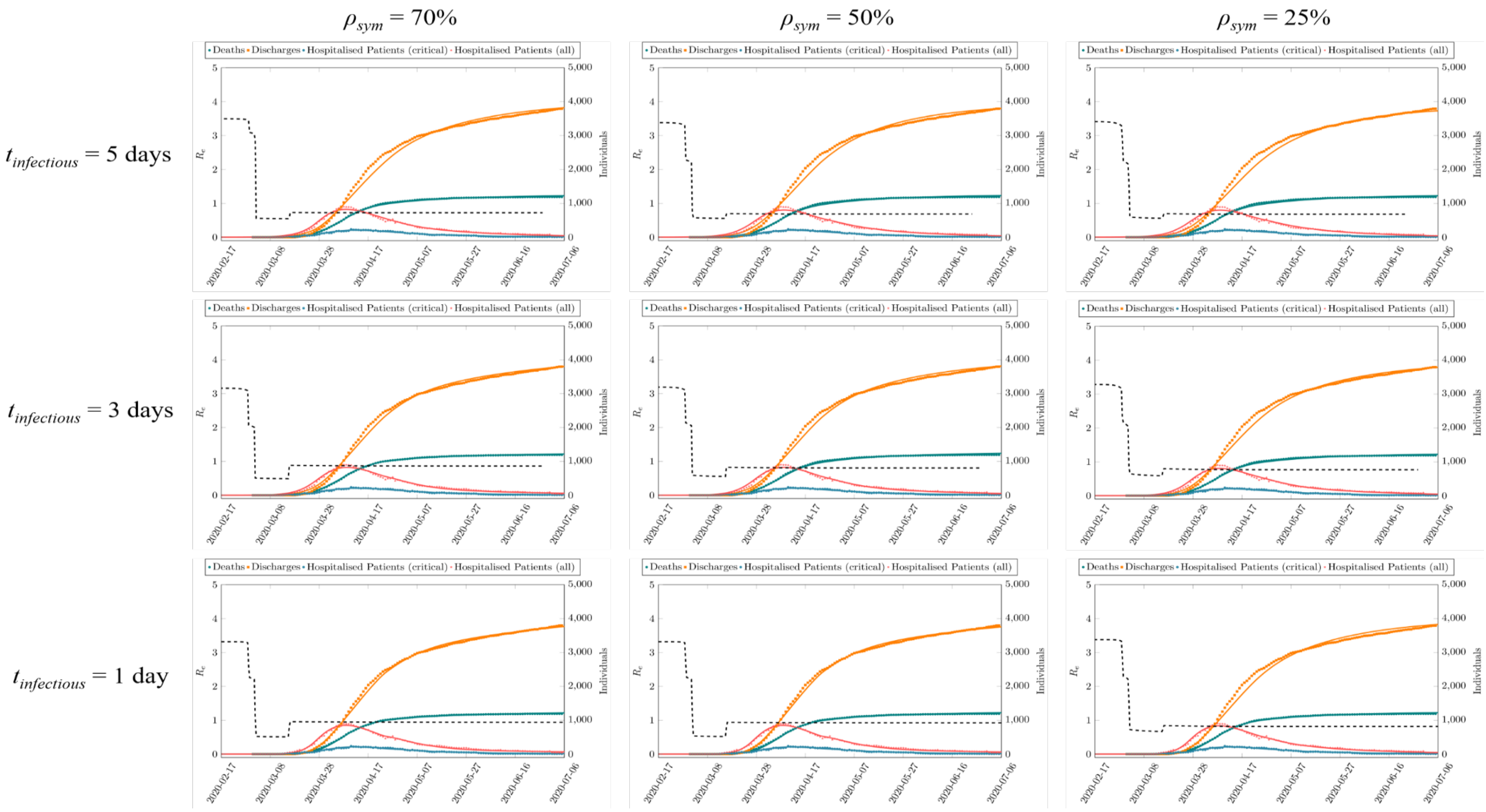

Figure S1: Calibration of the mathematical model to the number of hospitalised patients (all and in critical care beds), discharges and deaths in North East London NHS trusts, for varying combinations of infectious period and proportion of symptomatic infection. Observed data is given by coloured markers, and modelled data by solid lines. Effective reproduction number over time $R_e(t)$ (left axis) is displayed with dashed line.

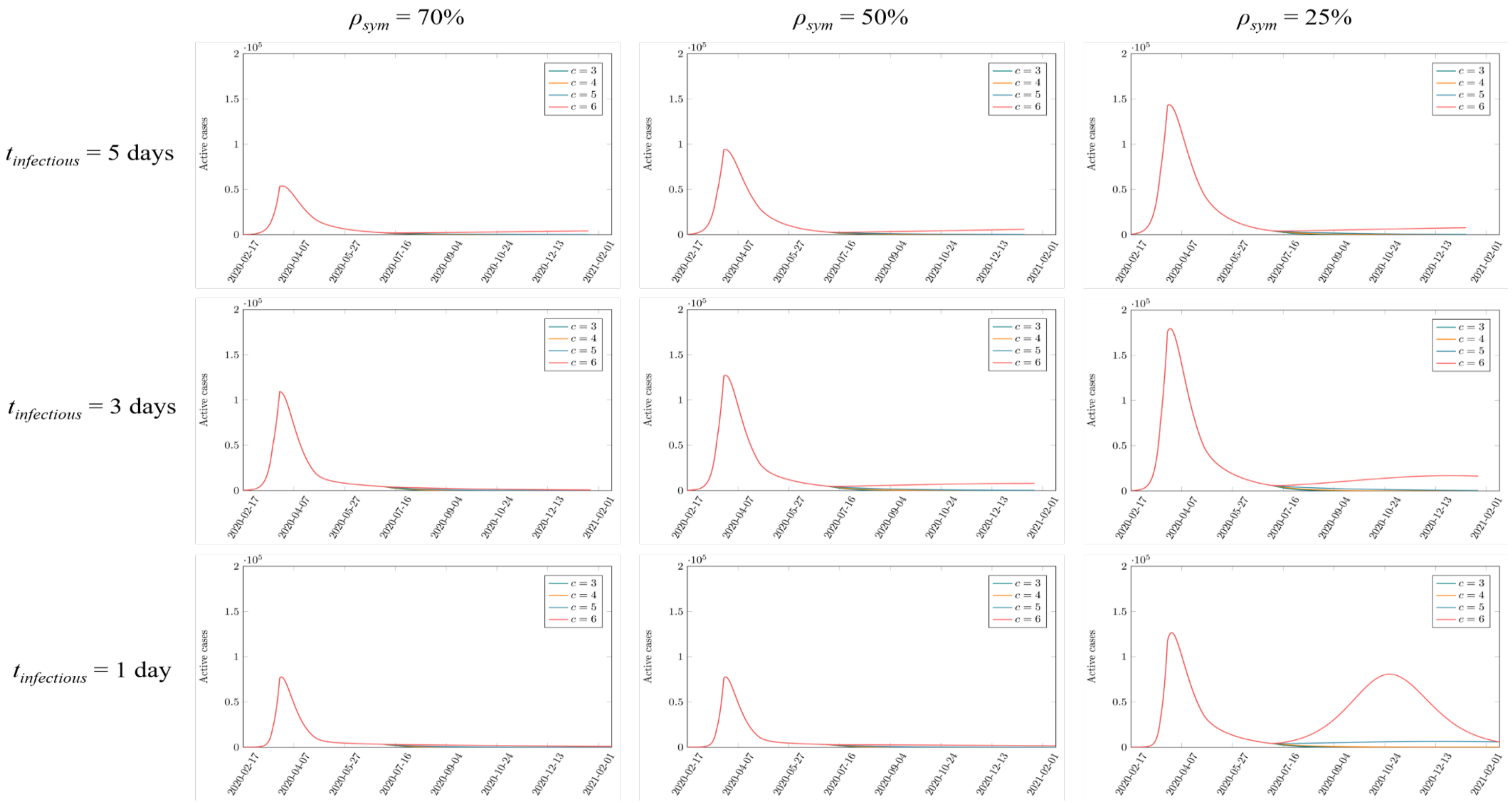

Figure S2: Projections of the mathematical model forecasting the number of COVID-19 cases over time for low numbers of average daily contacts, c = 3 to 6, for varying combinations of infectious period and proportion of symptomatic infection. We observed that a resurgence in the number of cases occurs if the proportion of symptomatic infection is lower and infectiousness period is shorter, with increased social interactions and c>5 or 6.

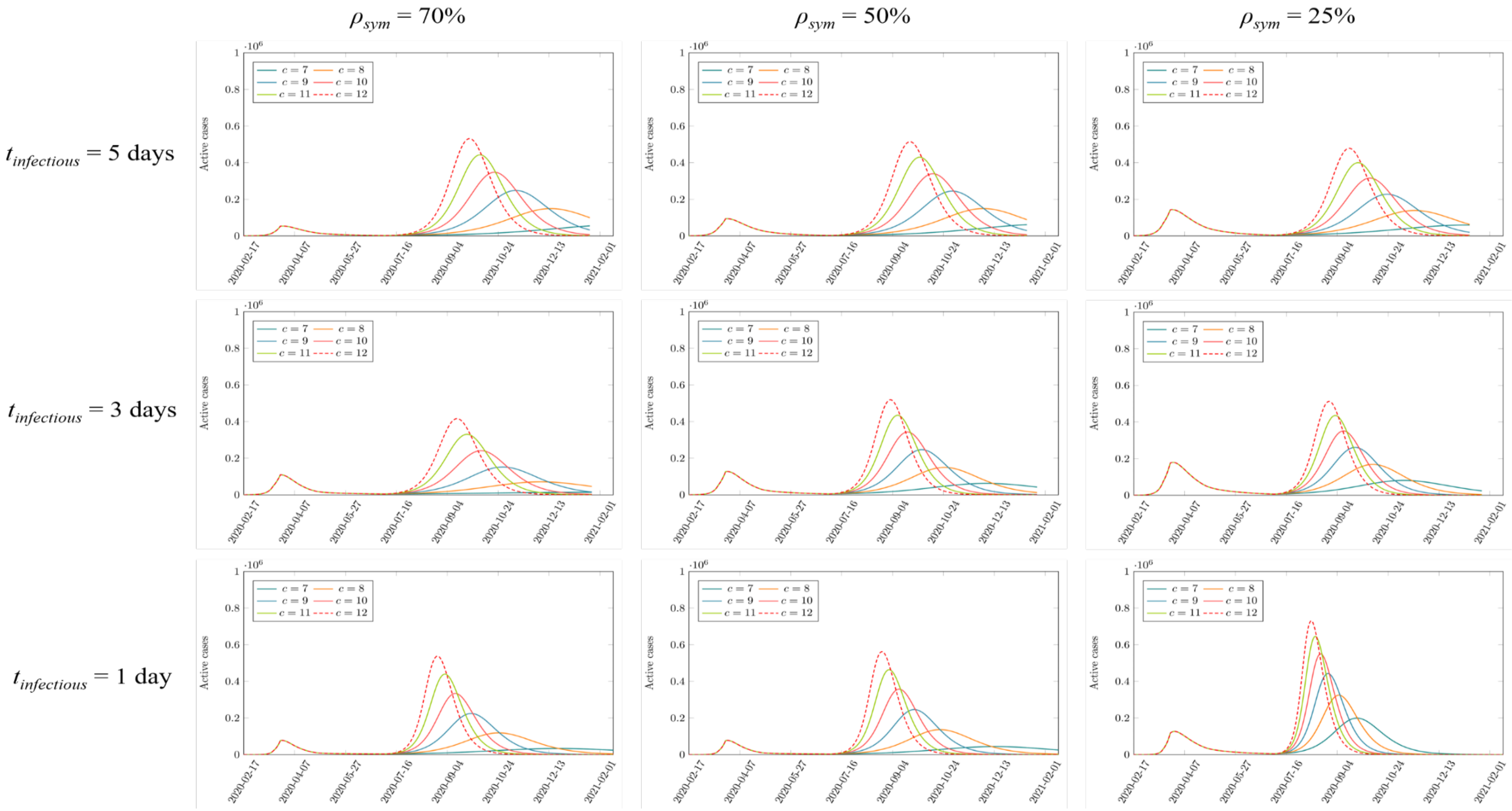

Figure S3: Projections of the mathematical model forecasting the number of COVID-19 cases over time for high numbers of average daily contacts, $c = 7$ to 12, for varying combinations of infectious period and proportion of symptomatic infection. We observed that a resurgence in the number of cases occurs for any combination of proportion of symptomatic infection and infectiousness period when average daily contacts are >6.

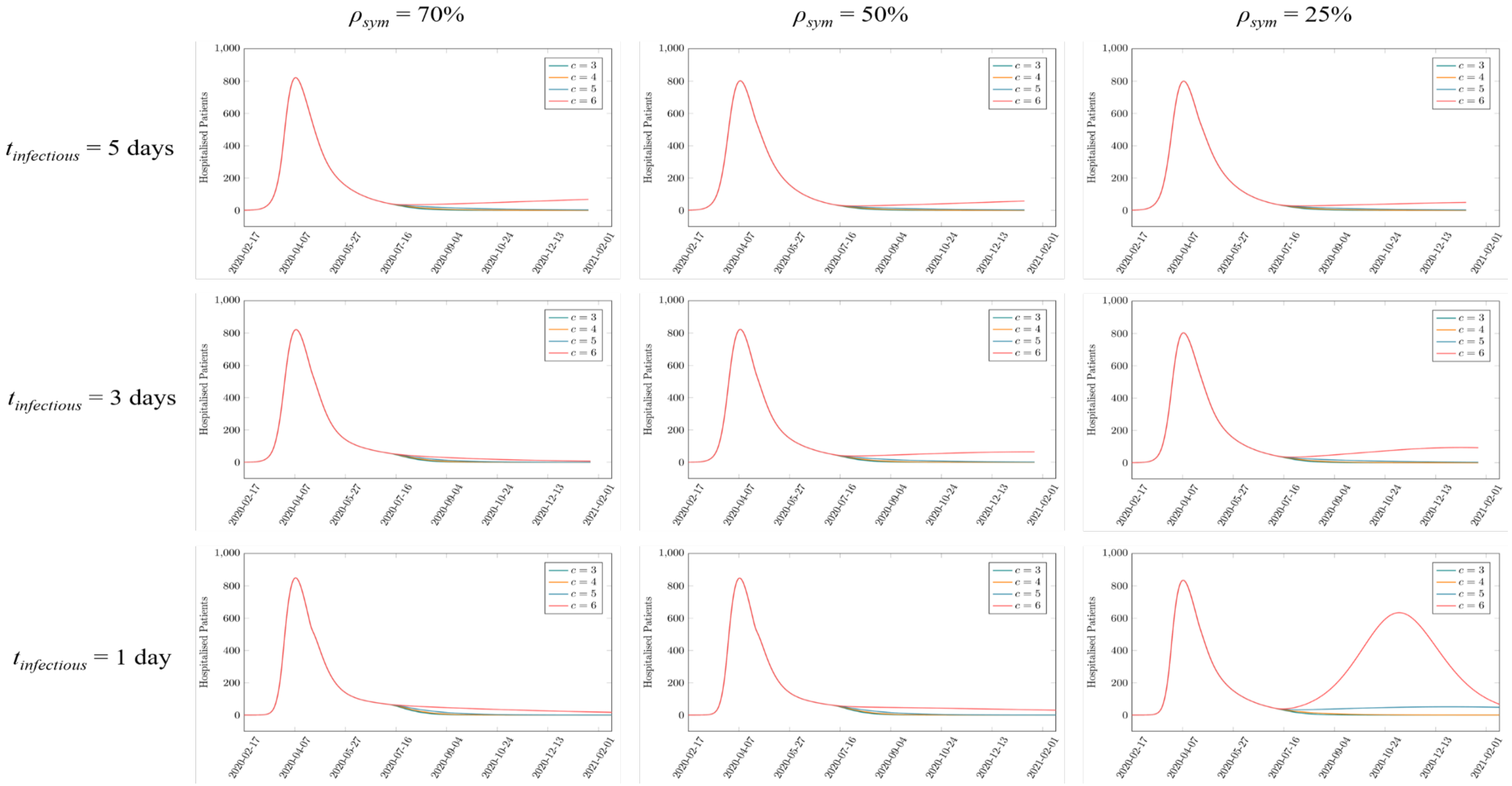

Figure S4: Projections of the mathematical model forecasting the number of patients hospitalised with COVID-19 over time for low numbers of average daily contacts, c = 3 to 6, for varying combinations of infectious period and proportion of symptomatic infection. We observed that a resurgence in the number of hospitalised patients occurs if the proportion of symptomatic infection is lower and the infectiousness period is shorter, with increased social interaction and c>5 or 6.

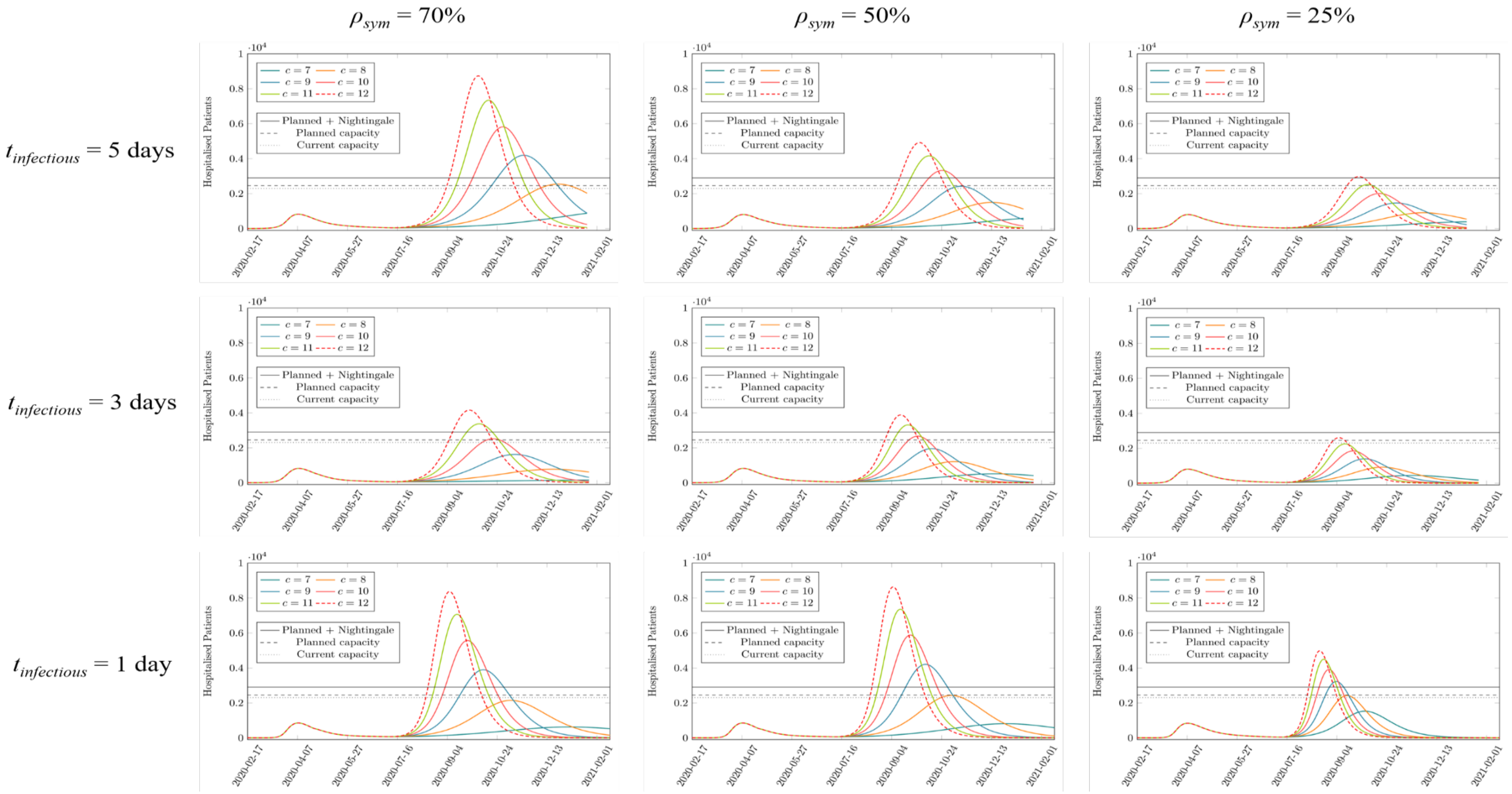

Figure S5: Projections of the mathematical model forecasting the number of patients hospitalised with COVID-19 over time for high numbers of average daily contacts, $c = 7$ to 12, for varying combinations of infectious period and proportion of symptomatic infection. We observed that a resurgence in the number of hospitalised patients occurs for any combination of proportion of symptomatic infection and infectiousness period when average daily contacts are >6, with acute bed capacity exceeded with larger c depending on scenarios.

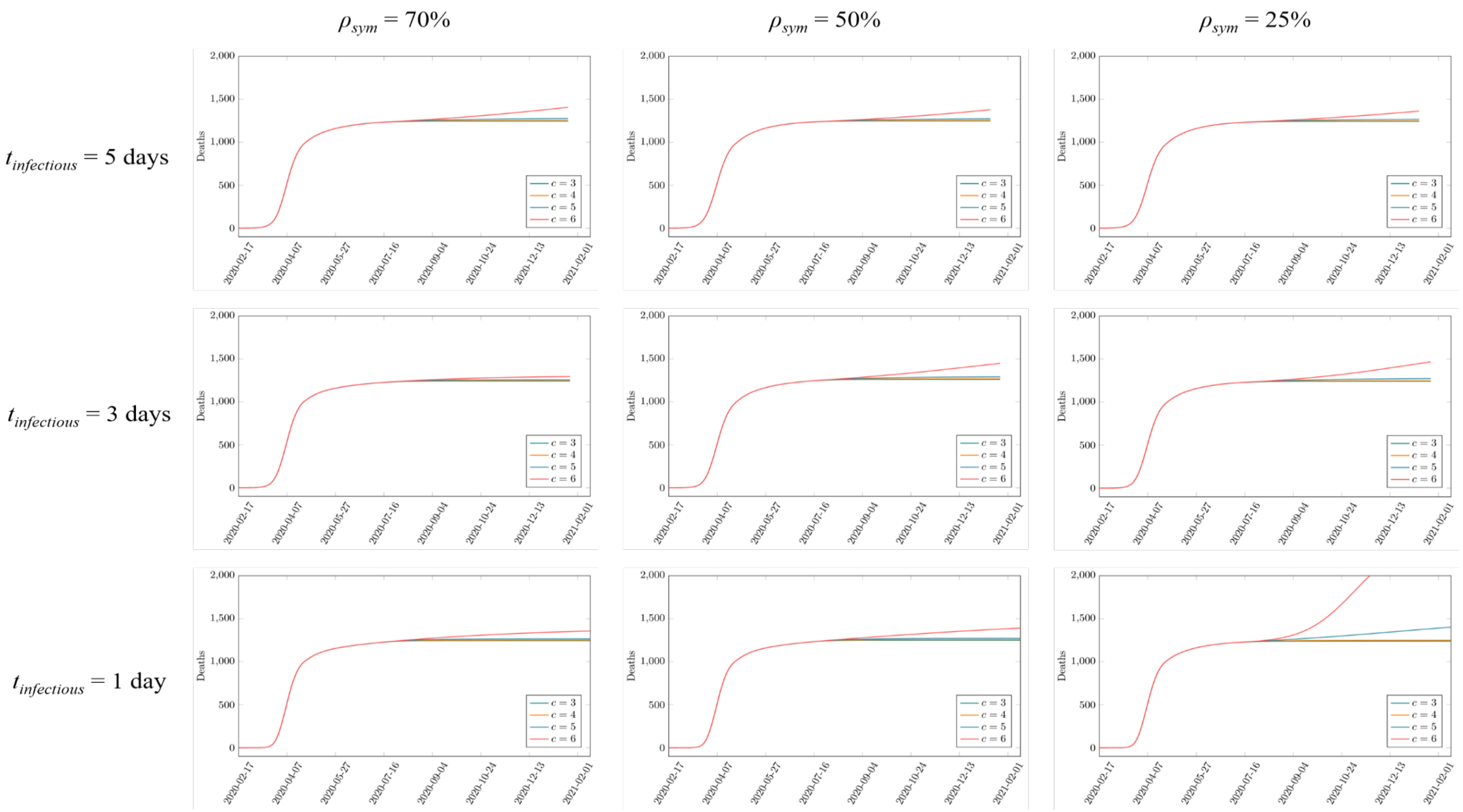

Figure S6: Projections of the mathematical model forecasting the cumulative number of deaths of patients hospitalised due to COVID-19 over time for low numbers of average daily contacts, c = 3 to 6, for varying combinations of infectious period and proportion of symptomatic infection. We observed that a resurgence of COVID-19 associated deaths occurs if the proportion of symptomatic infection is lower and infectiousness period is shorter, with increased social interaction and c>5 or 6.

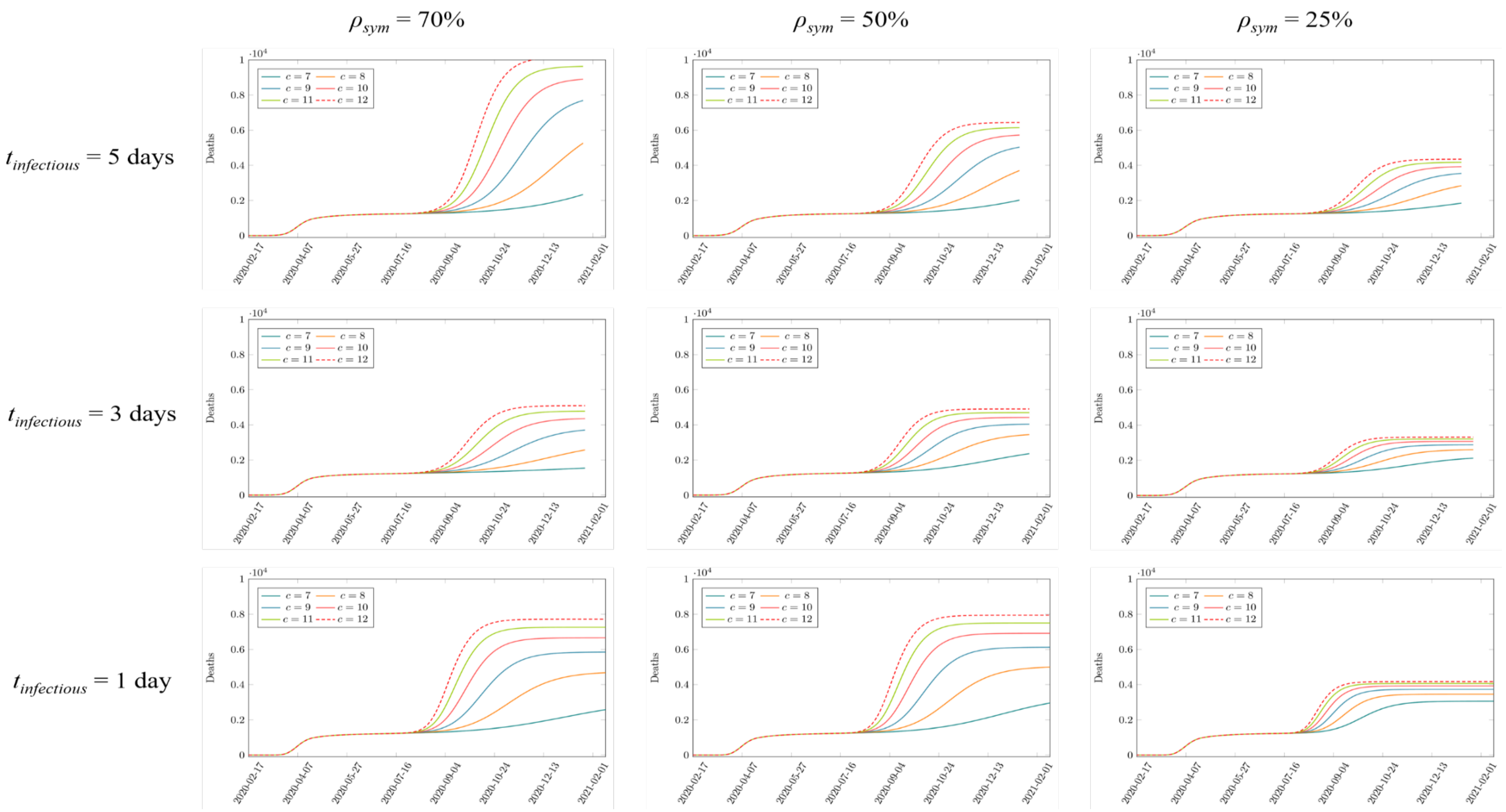

Figure S7: Projections of the mathematical model forecasting the cumulative number of deaths of patients hospitalised due to COVID-19 over time for high numbers of average daily contacts, $c = 7$ to 12, for varying combinations of infectious period and proportion of symptomatic infection. We observed that a resurgence in COVID-19 related deaths occurs for any combination of proportion of symptomatic infection and infectiousness period when average daily contacts are >6.

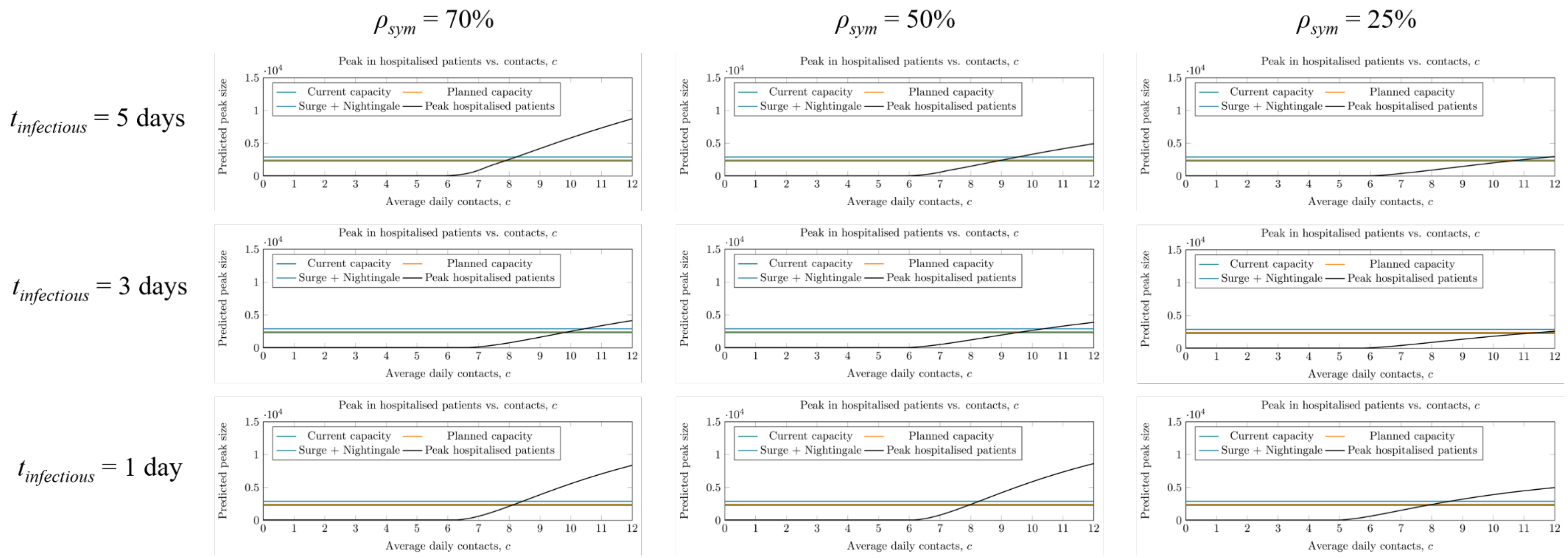

Figure S8: Predicted size of the secondary COVID-19 wave (black lines), in terms of the peak in all COVID-19 hospitalised patients, as a function of the daily number of contacts, c, from 4 July, for varying combinations of infectious period and proportion of symptomatic infection. Estimates for hospital capacity levels (coloured lines) in various scenarios are given for reference.

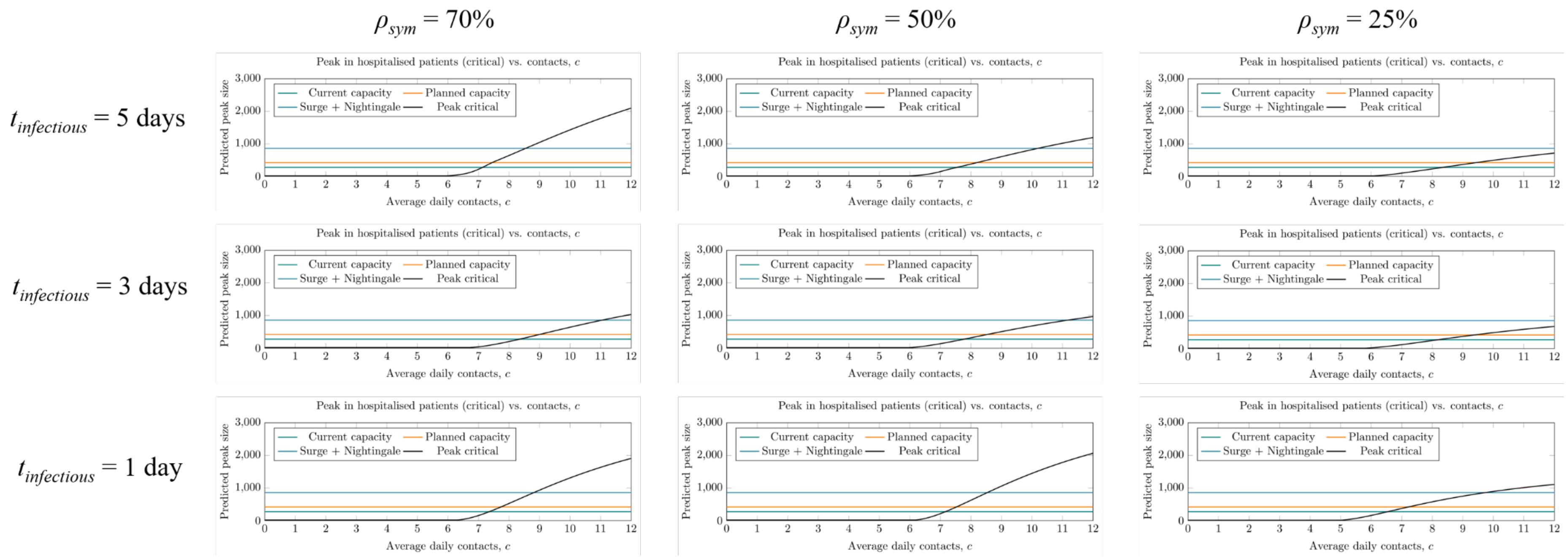

Figure S9: Predicted size of the secondary COVID-19 wave (black lines), in terms of the peak in critical care COVID-19 hospitalised patients, as a function of the daily number of contacts, c, from 4 July, for varying combinations of infectious period and proportion of symptomatic infection. Estimates for hospital capacity levels (coloured lines) in various scenarios are given for reference.

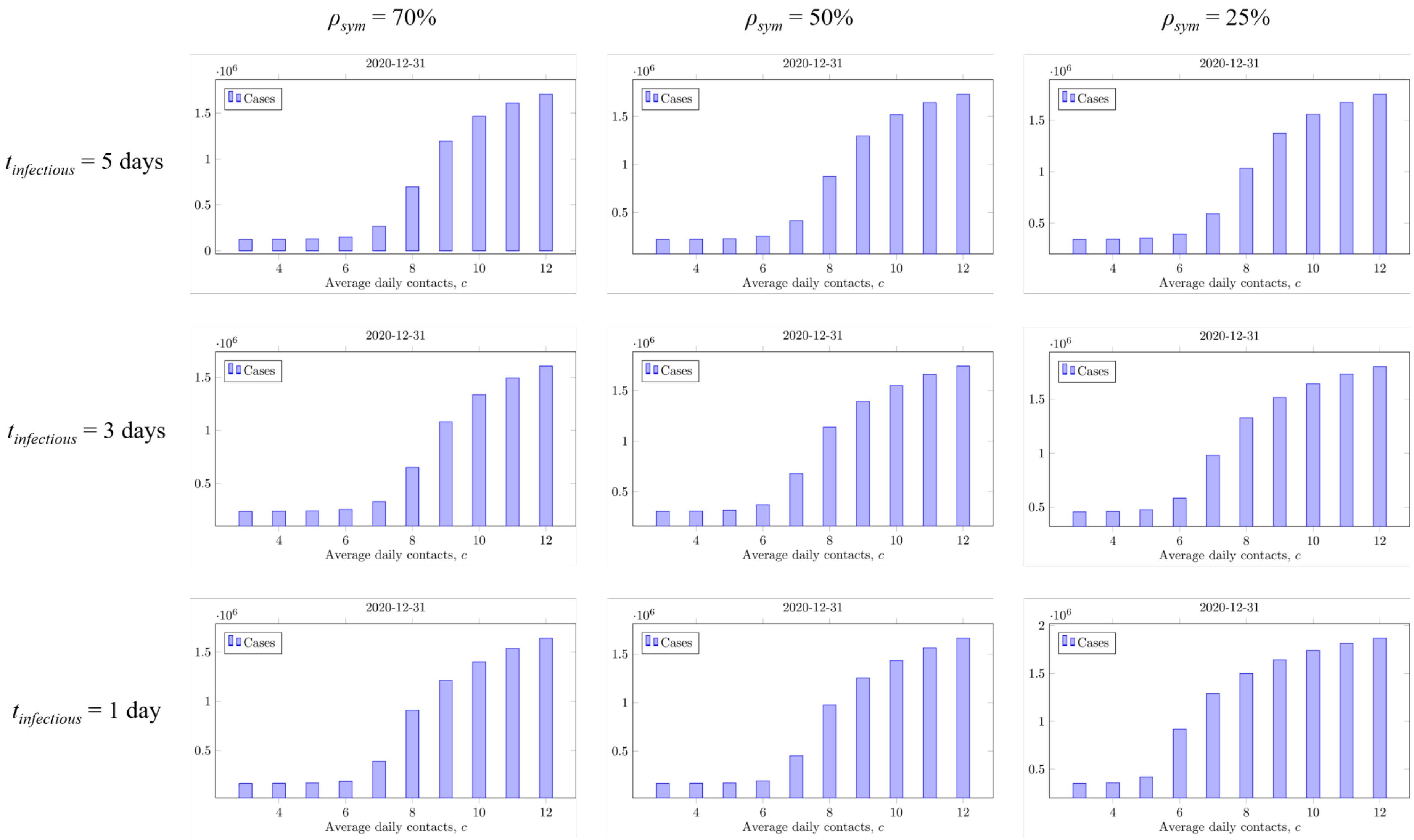

Figure S10: Cumulative number of COVID-19 cases as a function of the average number of daily contacts, c, from 4 July 2020, for varying combinations of infectious period and proportion of symptomatic infection. The cumulative totals for 2020 are considered, including forecasts up to 31 December 2020.

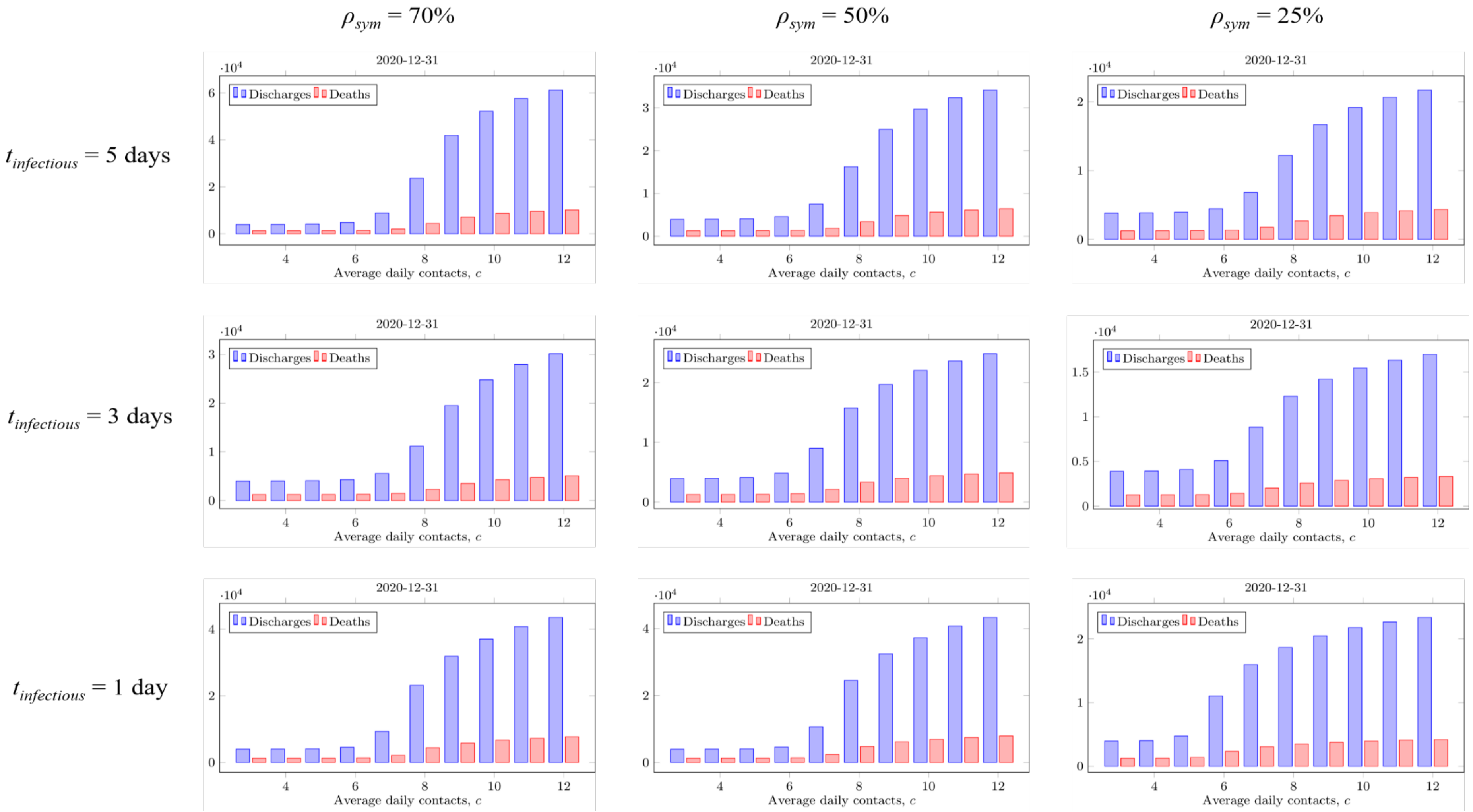

Figure S11: Cumulative number of hospital deaths and discharges from hospital for patients with COVID-19 as a function of the average number of daily contacts, c, from 4 July 2020, for varying combinations of infectious period and proportion of symptomatic infection. The cumulative totals for 2020 are considered, including forecasts up to 31 December 2020.

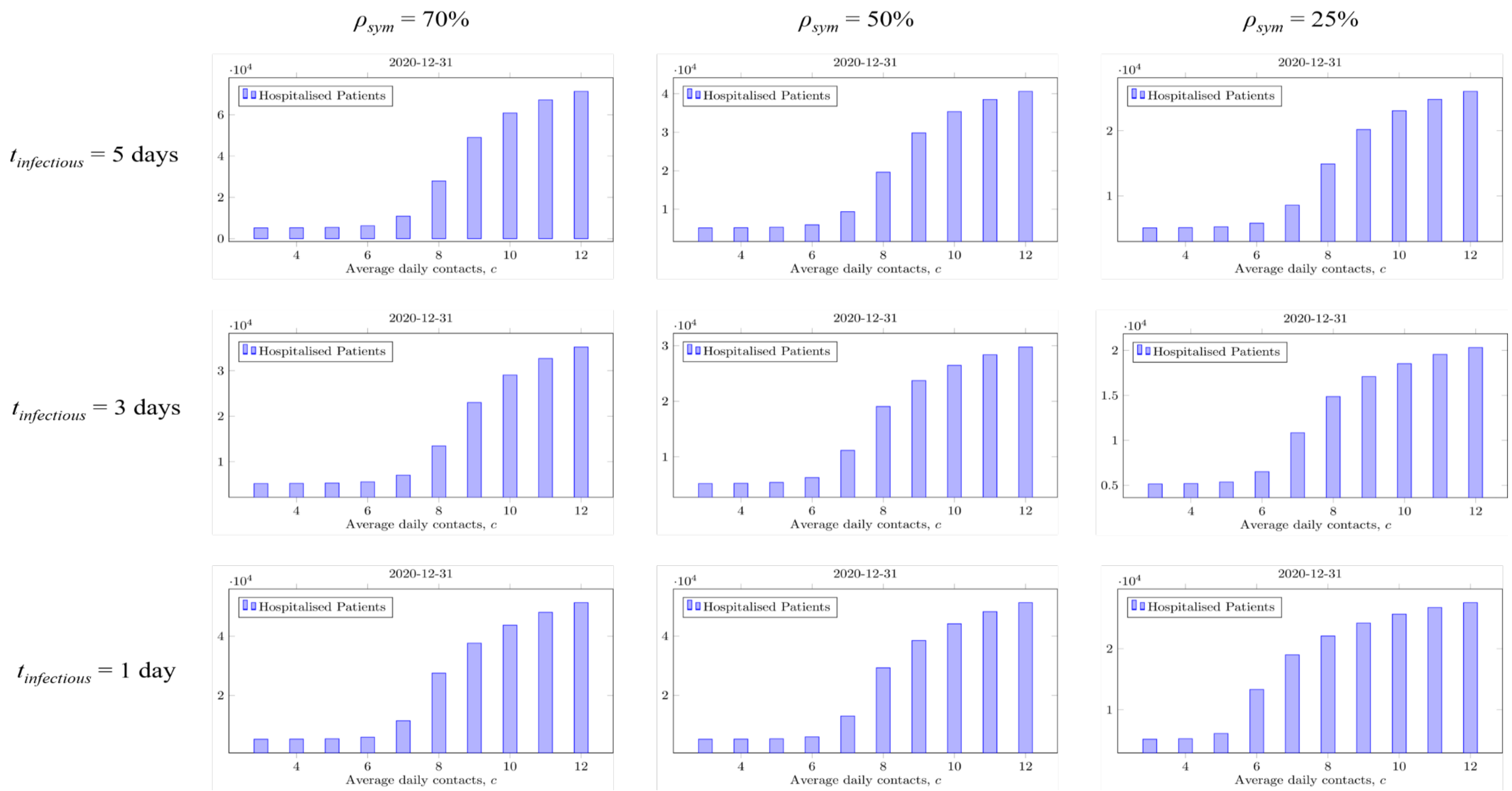

Figure S12: Cumulative number of patients hospitalised due to COVID-19 as a function of the average number of daily contacts, c, from 4 July 2020, for varying combinations of infectious period and proportion of symptomatic infection. The cumulative totals for 2020 are considered, including forecasts up to 31 December 2020.
